# Vitamin B supplementation enhances the efficacy of non-steroidal anti-inflammatory drugs in patients with painful foot and ankle conditions: A multicenter, prospective, randomized controlled trial

**DOI:** 10.1371/journal.pone.0336373

**Published:** 2025-11-13

**Authors:** Myung-Geun Song, Chul Hyun Park, Young-Rak Choi, Gi-Won Choi, Bom Soo Kim

**Affiliations:** 1 Department of Orthopaedic Surgery, Catholic Kwandong University International Saint Mary’s Hospital, Incheon, Republic of Korea; 2 Department of Orthopaedic Surgery, Yeungnam University College of Medicine, Daegu, Republic of Korea; 3 Department of Orthopedic Surgery, Asan Medical Center, School of Medicine, Ulsan University, Seoul, Korea; 4 Department of Orthopaedic Surgery, Ansan Hospital, Korea University College of Medicine, Ansan, Republic of Korea; 5 Department of Orthopaedic Surgery, Inha University Hospital, Incheon, Republic of Korea; Monash University, AUSTRALIA

## Abstract

**Objective:**

The management of foot disorders often involves anti-inflammatory drugs for chronic inflammation and pain. Combining anti-inflammatory drugs with vitamin B complex may have a synergistic effect, but its efficacy in foot disorders remains unclear. This study aimed to investigate the effects of combining vitamin B complex with anti-inflammatory drugs in patients with foot disorders.

**Methods:**

This study enrolled 201 patients aged ≥ 19 years with foot disorders of Achilles tendinitis, foot or ankle arthritis, Civinini-Morton syndrome (Morton’s neuroma), or plantar fasciitis from four hospitals in Korea between October 2020 and December 2021. The patients were randomized in a 1:1 ratio to receive either aceclofenac 100 mg twice daily alone (control group, n = 77) or aceclofenac 100 mg and vitamin B complex twice daily (experimental group, n = 79) for 4 weeks. Among 201 randomized participants, 156 patients(79 in the experimental group and 77 in the control group) completed all scheduled follow-up visits and were included in the final analysis. The primary outcome measure was pain as assessed using the 10-cm visual analog scale (VAS). The secondary outcome measures were health-related quality of life and foot/ankle outcomes as assessed using the EuroQol 5 Dimension (EQ-5D) questionnaire and the Foot and Ankle Outcome Score (FAOS), respectively.

**Results:**

VAS scores were more significantly reduced in the experimental group than in the control group, after 4 weeks (−2.87 ± 1.86 (from 6.95 ± 0.15 to 4.08 ± 0.17) vs. −0.91 ± 1.81 (from 5.99 ± 0.14 to 5.08 ± 0.21], p < 0.001). Similarly, improvements in the EQ-5D scores were significantly greater in the experimental group (0.079 ± 0.143 (from 0.69 ± 0.01 to 0.77 ± 0.01] vs 0.008 ± 0.111 (from 0.75 ± 0.01 to 0.76 ± 0.01], p = 0.001). Additionally, the increase in FAOS after treatment was significantly larger in the experimental group (9.94 ± 14.38 vs 2.97 ± 11.79, p = 0.001). Subgroup analysis revealed the combined therapy was particularly more effective in patients with chronic symptoms lasting over 3 months and in conditions like plantar fasciitis, foot or ankle arthritis, and Civinini-Morton syndrome.

**Conclusion:**

Combining vitamin B complex with anti-inflammatory drugs effectively reduces pain and improves function in patients with chronic foot disorders. This combination therapy offers a promising strategy for improving pain control and functional outcomes in orthopaedic outpatient settings.

**Trial registration:**

This study was registered at Clinical Research Information Service, Korea Centers for Disease Control and Prevention, Ministry of Health and WELFARE (Republic of Korea, https://cris.nih.go.kr/cris/search/detailSearchEn.do?seq=27976), a WHO International Clinical Trials Registry Platform primary register setup, on May 19,2020 under the registration number KCT0005035.

## Introduction

The foot and ankle are critical for weight-bearing, making them particularly vulnerable to strain. Particularly, chronic foot disorders, such as plantar fasciitis, achilles tendinitis, foot or ankle arthritis, and Civinini-Morton syndrome cause significant discomfort in daily life, and the associated pain can greatly diminish the patient’s quality of life [1]. These disorders often involve complex pain mechanisms, including mechanical, inflammatory, and neuropathic components. Current standard treatments include non-steroidal anti-inflammatory drugs (NSAIDs), physical therapy, orthoses, corticosteroid injections, and, in refractory cases, surgery [2–6].

Although analgesic anti-inflammatory drugs are commonly used to manage foot disorders, their long-term use is associated with adverse effects such as gastrointestinal bleeding, peptic ulcers, and increased cardiovascular risk, necessitating the development of safer and more effective treatment options [1,7,8]. Recent studies have suggested that vitamin B complex can enhance the effects of anti-inflammatory analgesics and reduce their side effects [9–12]. Vitamin B complex is known to protect the nervous system and regulate inflammatory responses, suggesting potential benefits for managing pain in chronic foot disorders [10,13]. However, its specific role in the pain associated with foot disorders remains unclear. Thus, this study aimed to evaluate the efficacy of combining anti-inflammatory drugs with vitamin B complex in alleviating pain and improving foot function in patients with chronic foot disorders

## Materials and methods

### Study design

This multicenter, prospective, randomized comparative trial was approved by the Institutional Review Board (IRB) of Inha University Hospital, Incheon, South Korea (IRB No: 2019-11-055) and was conducted according to the tenets of the Declaration of Helsinki. Written informed consent was obtained from all patients.

Patients were recruited from orthopedic outpatient clinics at four hospitals (Seoul Asan Medical Center, Inha University Hospital, Yeungnam University Hospital, and Korea University Ansan Hospital) between October 14, 2020 and December 15, 2021. Recruitment was conducted consecutively among eligible patients who visited for foot or ankle pain and met the inclusion criteria.

### Participants

Patients aged ≥19 years with foot disorder of Achilles tendinitis, foot or ankle arthritis, Civinini-Morton syndrome, or plantar fasciitis and with a visual analog scale (VAS) score of ≥4 cm were included. Patients were excluded if they had recent NSAID use, required surgical treatment, had systemic inflammatory diseases, or met other exclusion criteria. Randomization, patient enrollment, and treatment were conducted independently at each participating center in accordance with the standardized protocol. The inclusion and exclusion criteria for patient selection are summarized in Table 1.

**Table 1:** Inclusion and exclusion criteria for patient selection.

Inclusion criteria
1. Patients with four types of chronic inflammatory diseases of the foot and ankle diseases (foot or ankle arthritis, plantar fasciitis, Achilles tendinitis, Civinini-Morton syndrome).
2. Pain score of 4 cm or higher on a 10 cm visual analog scale (VAS).
3. Age ≥ 19 years
4. Requiring non-steroidal anti-inflammatory drugs (NSAIDs) for pain management.
**Exclusion criteria**
1. Initial pain score of ≤4 cm on a 10 cm VAS.
2. Use of NSAIDs or other analgesics within the last week.
3. Diagnosis of depression.
4. Requiring surgical treatment.
5. Presence of wounds in the affected area.
6. Current use of self-medicated vitamins.
7. Treatment for systemic autoimmune diseases.
8. Illiterate individuals or those who with difficultly understanding the study details.
9. Participation was discontinued by the researcher, with the approval from the research ethics committee, due to medication side effects.

### Pre study power analysis

A power analysis was performed based on a superiority trial design. In the DOLOR study by Mibielli et al. (2009) [14], the post-treatment VAS score was 12.9 ± 10 mm in the diclofenac plus B vitamins group and 20.1 ± 12 mm in the diclofenac monotherapy group. A superiority margin of 7 mm was defined. Using a two-sided alpha level of 0.05 and power (1–β) of 0.90, the required sample size was calculated using the formula: n = f(α, β) × 2 × σ²/d^2^, where f(α, β) = [Φ ⁻ ¹(α) + Φ ⁻ ¹(β)]^2^. A total of 73 subjects per group were required. Accounting for a 20% dropout rate, the final sample size was set at 100 participants per group (total n = 200).

### Randomization and blinding

Eligible participants were randomly assigned in a 1:1 ratio to either the experimental group (NSAIDs + vitamin B complex) or the control group (NSAIDs alone) using a centralized web-based electronic case report form (eCRF) system(http://www.proscore.kr). Block randomization was employed, stratified by study center to ensure balanced allocation across sites. Allocation concealment was maintained using opaque, sealed envelopes at each center.

This was an open-label randomized trial; no blinding was implemented for participants or outcome assessors. All procedures were conducted according to a standardized protocol applied uniformly across all participating institutions. Although this design may introduce expectation bias, the use of standardized protocols and smartphone-based self-reporting tools was intended to minimize bias. The absence of blinding should be considered a limitation.

### Intervention

The experimental group received aceclofenac 100 mg (Airtal®, Daewoong Pharmaceutical, Seoul, South Korea) twice daily and a vitamin B complex supplement (Impactamin® Power, Daewoong Pharmaceutical, Seoul, South Korea) twice daily for 4 weeks. The control group received aceclofenac 100 mg (Airtal®) twice daily only. The components of the vitamin B complex supplement (Impactamin® Power) used in the study are listed in Table 2.

**Table 2:** Ingredients of vitamin B complex tablet used in this study.

Ingredient	Amount
Thiamine (vit B1)	50 mg
Riboflavin (vit B2)	50 mg
Nicotinic acid amide	50 mg
Pantothenic acid	50 mg
Pyridoxine (vit B6)	50 mg
Folic acid	0.2 mg
Cyanocobalamin (vit B12)	50 mg
Choline bitartrate	50 mg
Inositol	50 mg
Biotin	0.05 mg
Zinc oxide	18.70 mg
Ascorbic acid	30 mg

Participants attended a total of five study visits: one baseline assessment and four weekly follow-up visits over the 4-week intervention period. All visits were conducted onsite at the outpatient clinics. Data on pain and functional outcomes were collected using standardized patient-reported outcome measures administered via smartphones.

### Diagnosis criteria

Diagnosis of the four target conditions were made by orthopedic specialists at each center based on clinical history, physical examination, and relevant imaging studies (e.g., X-rays, ultrasounds). Diagnosis criteria followed established definitions from the literature.

### Efficacy evaluation

Efficacy was evaluated using superiority testing of clinical assessment indices (VAS score [15], EuroQol 5 Dimensions [EQ-5D] score [16,17], and Foot and Ankle Outcome Score [FAOS] [18]). These indices were measured at five timepoints: at baseline immediately before treatment initiation and at 1, 2, 3, and 4 weeks thereafter.

#### Primary outcome.

Pain levels were assessed using the 10 cm-VAS, which featured a 10- cm line ranging from “no pain” (0 points) to “severe pain requiring treatment” (10 points). Patients quantified their level of pain by selecting a point on this line that best represented their pain intensity. [15].

#### Secondary outcome.

The EQ-5D score, which indicated overall health status, was assessed through a questionnaire, and scores ranged from 0 to 1, with 1 representing perfect health [16,17]. The FAOS, was also determined via the relevant questionnaire. Briefly, the FAOS is a self-assessment tool reflecting the condition of the foot and ankle, with scores ranging from 0 (extreme problems) to 100 (no problems) [18].

All evaluations conducted through patient surveys were performed via smartphones [19]. Additionally, the occurrence of any drug-related complications and adverse effects, such as digestive problems, diarrhea, constipation, rash, and nausea, reported during the patient visit was reported to the IRB after the researcher’s examination, and patients were excluded from the study as necessary.

### Pathology descriptions

This study included four common chronic foot and ankle disorders: plantar fasciitis, Achilles tendinitis, foot/ankle osteoarthritis, and Civinini-Morton syndrome.

Plantar fasciitis involves degeneration and inflammation of the plantar fascia due to repetitive microtrauma, leading to chronic heel pain, particularly during the first steps in the morning or after rest [2]. Achilles tendinitis is characterized by overuse-related degeneration of the Achilles tendon, resulting in localized pain and swelling in the posterior ankle, often worsened by activity [5]. Foot and ankle osteoarthritis is a degenerative joint disease marked by cartilage loss, joint space narrowing, and subchondral bone changes, commonly affecting the tibiotalar or subtalar joints. It leads to pain, stiffness, and limited mobility, especially in weight-bearing activities [6]. Civinini-Morton syndrome (formerly Morton’s neuroma) is a compressive neuropathy of the interdigital nerve, typically causing forefoot pain and numbness between the third and fourth toes. Symptoms are often aggravated by tight footwear or prolonged standing. Although mechanical offloading with shoe modifications and custom orthotics is considered the mainstay of conservative treatment [4], NSAIDs are also commonly prescribed in clinical practice to alleviate inflammation and pain in the early stages, particularly when neuritic symptoms coexist with soft tissue irritation [3].

### Statistical analysis

All results were presented as mean ± standard deviation or frequencies. The Student’s t-test was used for continuous variables, whereas Pearson’s chi-square or Fisher’s exact test was used for categorical variables. All statistical analyses were conducted using the Statistical Package for Social Sciences (SPSS) statistical software (version 23.0, IBM Corp., Armonk, NY, USA). Statistical significance was set at p < 0.05.

Although the study was conducted at multiple centers, potential heterogeneity across study sites was minimized through stratified randomization by center, the use of a standardized intervention protocol, and uniform outcome assessment using self-administered smartphone-based PROMs. Therefore, a random-effects model was not applied, as between-center variability was considered negligible. Missing data were handled using a complete case analysis approach, whereby only participants with complete data across all timepoints for the primary and secondary outcomes were included in the final analyses. Analyses were conducted based on the intention-to-treat(ITT) principle, and participants who completed all scheduled evaluations were included in the final analysis.

To compare treatment effects, both adjucted and unadjusted analyses were conducted. For primary and secondary outcome measures(VAS, EQ-5D, and FAOS), analysis of covariance(ANCOVA) was used to compared Week 4 outcome between groups while adjusting for baseline values. Linear mixed-effects models(LMMs) were used to evaluate repeated-measures outcomes across the 4-week period, including fixed effects for group, time, and group x time interaction.

Additionally, for descriptive clarity, mean change scores(Δ values) from baseline to each timepoint were calculated for VAS, EQ-5D, and FAOS, and compared between groups using independent-samples t-tests. These results are presented in Tables 5–7. Subgroup analyses based on disease type and symptom duration were also conducted using independent-samples t-tests based on unadjusted ΔVAS values (Tables 8–10). These subgroup comparisons were exploratory and not adjusted for baseline differences. The study protocol, statistical plan, and complete dataset are available in the supporting information (S1 Appendix).

**Table 3:** Comparison of baseline characteristics between the experimental and control groups.

Characteristic	Experimental group (aceclofenac + vitB complex, n = 79)	Control group (aceclofenac alone, n = 77)	p-Value
**Age(years)**	55.53 ± 13.83	56.97 ± 12.68	0.489
**Sex (female), n (%)**	47 (59.4)	47 (61.0)	0.844
**Height (cm)**	163.36 ± 8.53	163.06 ± 8.46	0.132
**Weight (kg)**	67.30 ± 12.08	64.67 ± 12.77	0.751
**Patient’s distribution by disease, n (%)**			0.711
** Achilles tendinitis**	7 (8.9)	11 (14.2)	
** Ankle/foot osteoarthritis**	21 (26.6)	20 (26.0)	
** Civinini-Morton syndrome**	23 (29.1)	23 (29.9)	
** Plantar fasciitis**	28 (35.4)	23 (29.9)	
**Disease duration, n (%)**			0.796
** Less than 3 months**	7 (8.9)	11 (14.2)	
** 3–6 Months**	15 (19.0)	13 (16.9)	
** 6 Months to 1 year**	14 (17.7)	13 (16.9)	
** 1–2 Years**	16 (20.2)	18 (23.3)	
** Over 2 years**	27 (34.1)	22 (28.5)	
**Alcohol consumption history, n (%)**			0.999
** Alcoholic without neuropathy**	1 (1.3)	1 (1.3)	
** Non-alcoholic**	47 (59.5)	46 (59.7)	
** Social drinker**	31 (39.2)	30 (39.0)	
**Diabetes mellitus, n (%)**			0.746
** Non-DM**	72 (91.1)	69 (89.6)	
** DM without neuropathy**	7 (8.9)	8 (10.4)	
**Smoking history, n (%)**			0.556
** Current smoker**	10 (12.7)	11 (14.2)	
** Previous smoker**	60 (76.0)	61 (79.2)	
** Non-smoker**	9 (11.3)	5 (6.5)	

**Table 4:** Comparison of outcome measures between the two groups.

Outcome measure		Experimental group (aceclofenac + vitB complex, n = 79)	Control group (aceclofenac alone, n = 77)	p-Value*
**VAS**	Baseline	6.95 ± 0.15	5.99 ± 0.14	0.008
	Visit 1	5.51 ± 0.20	5.40 ± 0.19	0.295
	Visit 2	5.29 ± 0.18	5.04 ± 0.18	0.143
	Visit 3	4.72 ± 0.17	4.94 ± 0.19	0.865
	Visit 4	4.08 ± 0.17	5.08 ± 0.21	<0.001
p-Value		<0.001	<0.001	
**EQ-5D**	Baseline	0.69 ± 0.01	0.75 ± 0.01	0.152
	Visit 1	0.72 ± 0.01	0.74 ± 0.01	0.459
	Visit 2	0.76 ± 0.01	0.77 ± 0.01	0.545
	Visit 3	0.75 ± 0.01	0.77 ± 0.01	0.602
	Visit 4	0.77 ± 0.01	0.76 ± 0.01	0.001
p-Value		<0.001	0.509	
**FAOS**	Baseline	55.68 ± 1.95	64.88 ± 1.88	0.462
	Visit 4	65.62 ± 1.56	66.73 ± 1.62	0.030
p-Value		<0.001	0.030	

VAS, visual analog scale; EQ-5D, EuroQol 5 Dimensions; FAOS, Foot and Ankle Outcome Score.

*Values are adjusted means and standard errors based on ANCOVA and linear mixed-effects modeling. Between-group comparisons were adjusted for baseline values using ANCOVA for week 4, and temporal trends were assessed using linear mixed-effects modeling.

**Table 5:** Change in VAS Scores at Each Timepoint: Comparison Between Experimental and Control Groups.

VAS score	Measurement timepoint	Experimental group (aceclofenac + vitB complex, n = 79)	Control group (aceclofenac alone, n = 77)	p-Value
**Δ VAS**	Visit 1	−1.44 ± 0.66	−0.58 ± 1.51	0.001
	Visit 2	−1.66 ± 1.64	−0.95 ± 1.58	0.007
	Visit 3	−2.23 ± 1.78	−1.05 ± 1.82	0.000
	Visit 4	−2.87 ± 1.86	−0.91 ± 1.81	0.000

**Δ VAS, difference in VAS between two measurement timepoints.**

VAS, visual analog scale.

**Table 6:** Change in EQ-5D at Each Timepoint: Comparison Between Experimental and Control Groups.

EQ-5D score	Measurement timepoint	Experimental group (aceclofenac + vitB complex, n = 79)	Control group (aceclofenac alone, n = 77)	p-Value
**Δ EQ-5D**	Visit 1	0.029 ± 0.141	−0.013 ± 0.099	0.032
	Visit 2	0.065 ± 0.139	0.013 ± 0.105	0.01
	Visit 3	0.057 ± 0.147	0.013 ± 0.110	0.037
	Visit 4	0.079 ± 0.143	0.008 ± 0.111	0.001

ΔEQ-5D, difference in EQ-5D scores between two measurement timepoints.

EQ-5D, EuroQol 5 Dimensions.

**Table 7:** Change in FAOS: Comparison Between Experimental and Control Groups.

FAOS	Measurement timepoint	Experimental group (aceclofenac + vitB complex, n = 79)	Control group (aceclofenac alone, n = 77)	p-Value
**Δ FAOS**	Visit 4	9.94 ± 14.38	2.97 ± 11.79	0.001

ΔFAOS, difference in FAOS between two measurement timepoints.

FAOS, Foot and Ankle Outcome Score.

**Table 8:** Between-group comparison of change in VAS score by foot condition.

Condition	Group	Baseline	Visit 1	Visit 2	Visit 3	Visit 4	p-Value
**Achilles tendinitis**	Experimental group (aceclofenac + vitB complex, n = 7)	7.14 ± 1.68	6.14 ± 1.68	5.29 ± 1.98	5.00 ± 1.83	4.43 ± 2.23	0.004*
	Control group (aceclofenac alone, n = 11)	6.64 ± 1.69	5.36 ± 1.86	5.00 ± 2.09	5.36 ± 2.25	5.82 ± 2.44	0.347
**p-Value**		0.543	0.382	0.777	0.725	0.242	
**Ankle/foot osteoarthritis**	Experimental group (aceclofenac + vitB complex, n = 21)	6.81 ± 1.44	5.19 ± 2.23	4.90 ± 1.99	4.81 ± 1.72	4.19 ± 1.56	<0.001*
	Control group (aceclofenac alone, n = 20)	5.55 ± 1.32	5.35 ± 1.57	5.20 ± 1.47	4.85 ± 2.18	5.25 ± 1.71	0.201
**p-Value**		0.006	0.793	0.595	0.948	0.045	
**Civinini-Morton syndrome**	Experimental group (aceclofenac + vitB complex, n = 23)	6.87 ± 1.74	5.70 ± 2.09	4.70 ± 1.79	4.39 ± 1.64	4.61 ± 2.01	<0.001*
	Control group (aceclofenac alone, n = 23)	5.83 ± 1.19	5.35 ± 1.53	4.70 ± 1.79	4.39 ± 1.64	4.61 ± 2.02	0.007*
**p-Value**		0.022	0.524	0.177	0.855	0.170	
**Plantar fasciitis**	Experimental group (aceclofenac + vitB complex,n = 28)	7.07 ± 1.27	5.43 ± 2.32	5.50 ± 1.55	4.79 ± 2.01	4.07 ± 1.76	<0.001*
	Control group (aceclofenac,n = 23)	6.22 ± 1.20	5.52 ± 1.28	5.26 ± 1.21	5.35 ± 1.47	5.04 ± 1.61	0.002*
**p-Value**		0.018	0.864	0.550	0.268	0.047	

**Table 9:** Between-group comparison of change in VAS score by disease duration.

Duration	Group	Baseline	Visit 1	Visit 2	Visit 3	Visit 4	p-Value
**Less than 3 months**	Experimental group (aceclofenac + vitB complex, n = 7)	6.29± 1.38	4.86 ± 1.57	4.43 ± 1.71	4.00 ± 1.53	3.71 ± 1.38	0.006*
	Control group (aceclofenac alone, n = 11)	6.45 ± 1.51	5.55 ± 1.81	4.73 ± 1.85	4.18 ± 1.78	4.27 ± 2.15	0.031*
**p-Value**		0.814	0.421	0.736	0.827	0.551	
**3–6 Months**	Experimental group (aceclofenac + vitB complex, n = 15)	6.93 ± 1.62	5.47 ± 1.84	5.40 ± 1.72	4.53 ± 1.56	3.67 ± 1.45	<0.001*
	Control group (aceclofenac alone, n = 13)	6.31± 1.18	5.62 ± 1.66	5.69 ± 1.49	5.85 ± 1.41	5.54 ± 1.89	0.156
**p-value**		0.261	0.826	0.638	0.019	0.007	
**6 Months to 1 year**	Experimental group (aceclofenac + vitB complex, n = 14)	7.29 ± 1.38	5.86 ± 2.74	5.21 ± 1.89	4.36 ± 2.31	4.07 ± 2.02	<0.001*
	Control group (aceclofenac alone, n = 13)	5.31 ± 1.18	4.85 ± 1.35	4.31 ± 1.75	3.77 ± 2.09	4.15 ± 2.12	0.521
**p-Value**		0.001	0.241	0.209	0.495	0.918	
**1–2 Years**	Experimental group (aceclofenac + vitB complex, n = 16)	7.25 ± 1.44	5.44 ± 2.63	5.63 ± 2.34	5.19 ± 1.97	4.31 ± 1.96	<0.001*
	Control group (aceclofenac alone, n = 18)	5.78 ± 1.06	5.56 ± 1.50	5.22 ± 1.35	5.22 ± 1.87	5.56 ± 1.54	0.016*
**p-Value**		0.002	0.872	0.537	0.958	0.047	
**Over 2 years**	Experimental group (aceclofenac + vitB complex, n = 27)	6.78 ± 1.50	5.56 ± 1.91	5.30 ± 1.20	4.93 ± 1.59	4.26 ± 1.53	0.000
	Control group (aceclofenac alone, n = 22)	6.14 ± 1.52	5.41 ± 1.33	5.09 ± 1.54	5.23 ± 1.63	5.36 ± 1.73	0.000
**p-Value**		0.146	0.762	0.603	0.518	0.022	

**Table 10:** Between-group comparison of amount of change in VAS score at 4 weeks by condition and duration.

VAS score change	Experimental group (aceclofenac + vitB complex, n = 79)	Control group (aceclofenac alone, n = 77)	p-Value
**Overall**	−2.87 ± 1.86	−0.91 ± 1.81	0.000
**Condition**			
** Achilles tendinitis**	−2.71 ± 2.69	−0.82 ± 2.75	0.17
** Ankle/foot osteoarthritis**	−2.62 ± 1.83	−0.30 ± 1.03	0.000
** Civinini-Morton syndrome**	−3.00 ± 2.00	−1.22 ± 1.95	0.004
** Plantar fasciitis**	−3.00 ± 1.61	−1.17 ± 1.64	0.000
**Disease duration**			
** Less than 3 months**	−2.57 ± 1.61	−2.18 ± 2.89	0.751
** 3–6 Months**	−3.27 ± 2.18	−0.77 ± 1.83	0.003
** 6 Months to 1 year**	−3.21 ± 1.42	−1.15 ± 1.40	0.001
** 1–2 Years**	−2.94 ± 1.48	−0.22 ± 1.43	0.000
** Over 2 years**	−2.52 ± 2.15	−0.77 ± 1.37	0.002

## Results

### Patient characteristics

**Fig 1:**
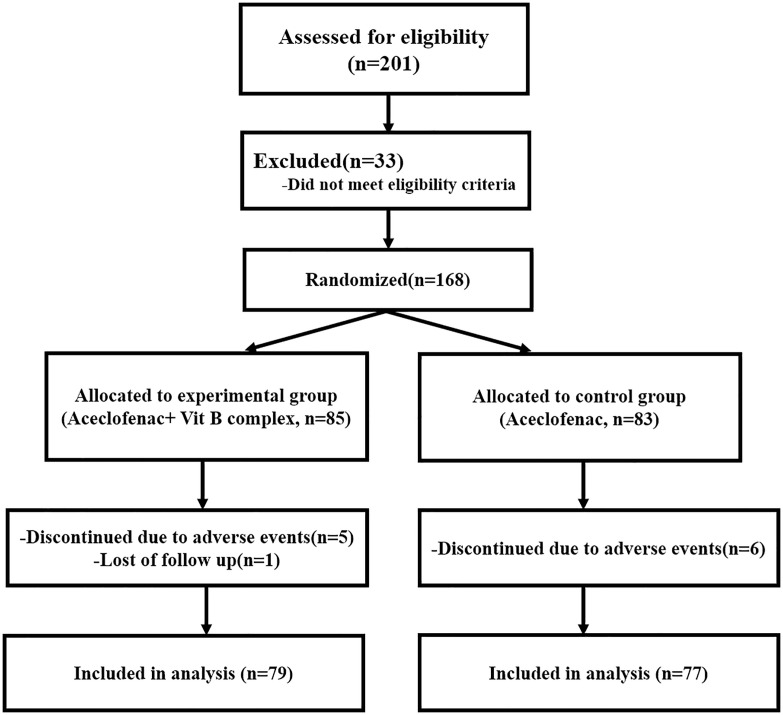
CONSORT flow diagram of patient enrollment, allocation, follow-up, and analysis.

Among the 201patients initially enrolled, 33 were excluded due to not meeting inclusion criteria or withdrawal of consent, resulting in 168 patients who were randomized into the two study groups. Of these, 85 were allocated to the experimental group (Aceclofenac and vitamin B complex) and 83 to the control group (Aceclofenac alone). One patient in the experimental group completed only the baseline assessment and did not return for any follow-up visits, and was therefore excluded from repeated-measures analyses. As a result, 79 and 77 patients were included in the final analyses of the experimental and control groups, respectively. The first study patient was enrolled on October 14, 2020 and the final data collection ended on December 15, 2021. The flow of participants is detailed in Fig 1.

The average patients ages in the experimental and control groups were 55.53 ± 13.83 years and 56.97 ± 12.68 years, respectively. Sex, height, weight, alcohol consumption, presence of diabetes, or smoking history was not significantly different between the groups. There were also no significant between group differences in the distribution of foot disorders or condition duration (Table 3). Detailed baseline characteristics of both groups are provided in the Supporting information (S1 Table).

### Clinical treatment efficacy

After adjusting for baseline values using ANCOVA and linear mixed-effects models, all three outcome measures showed greater improvement in the experimental group compared to the control group.

**Fig 2:**
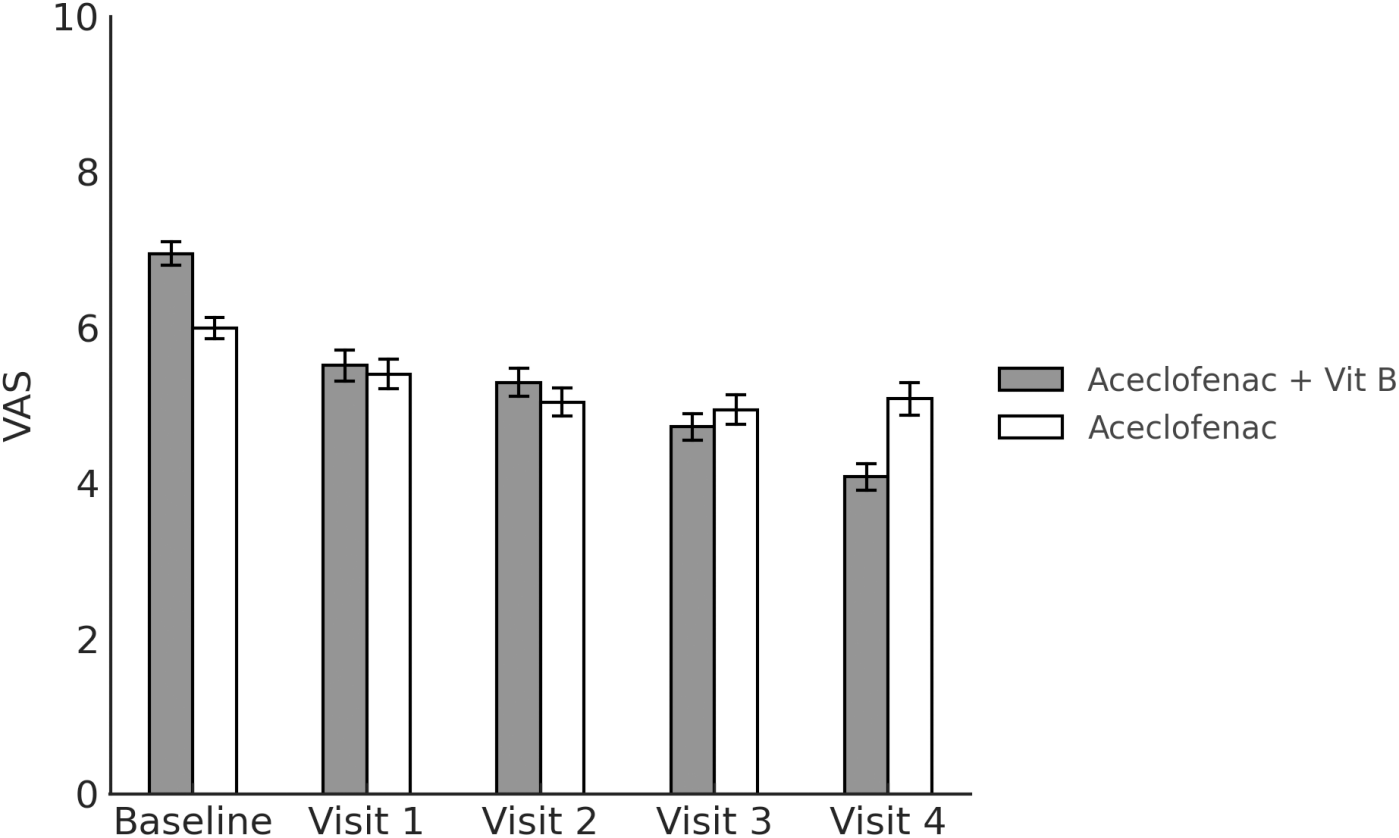
Visual analog scale score at each measurement timepoint.

**Fig 3:**
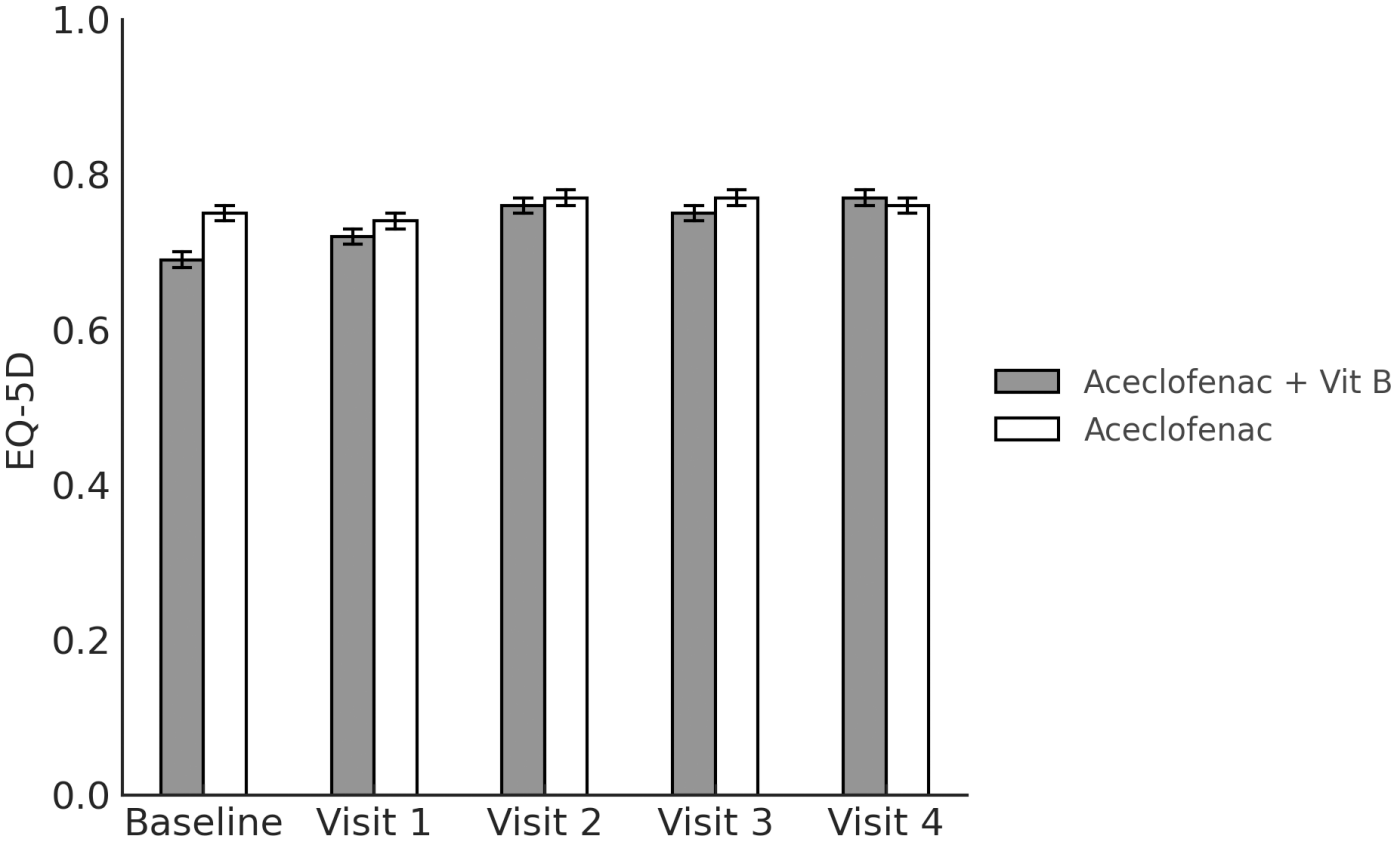
EuroQol 5 Dimension score at each measurement timepoint.

**Fig 4:**
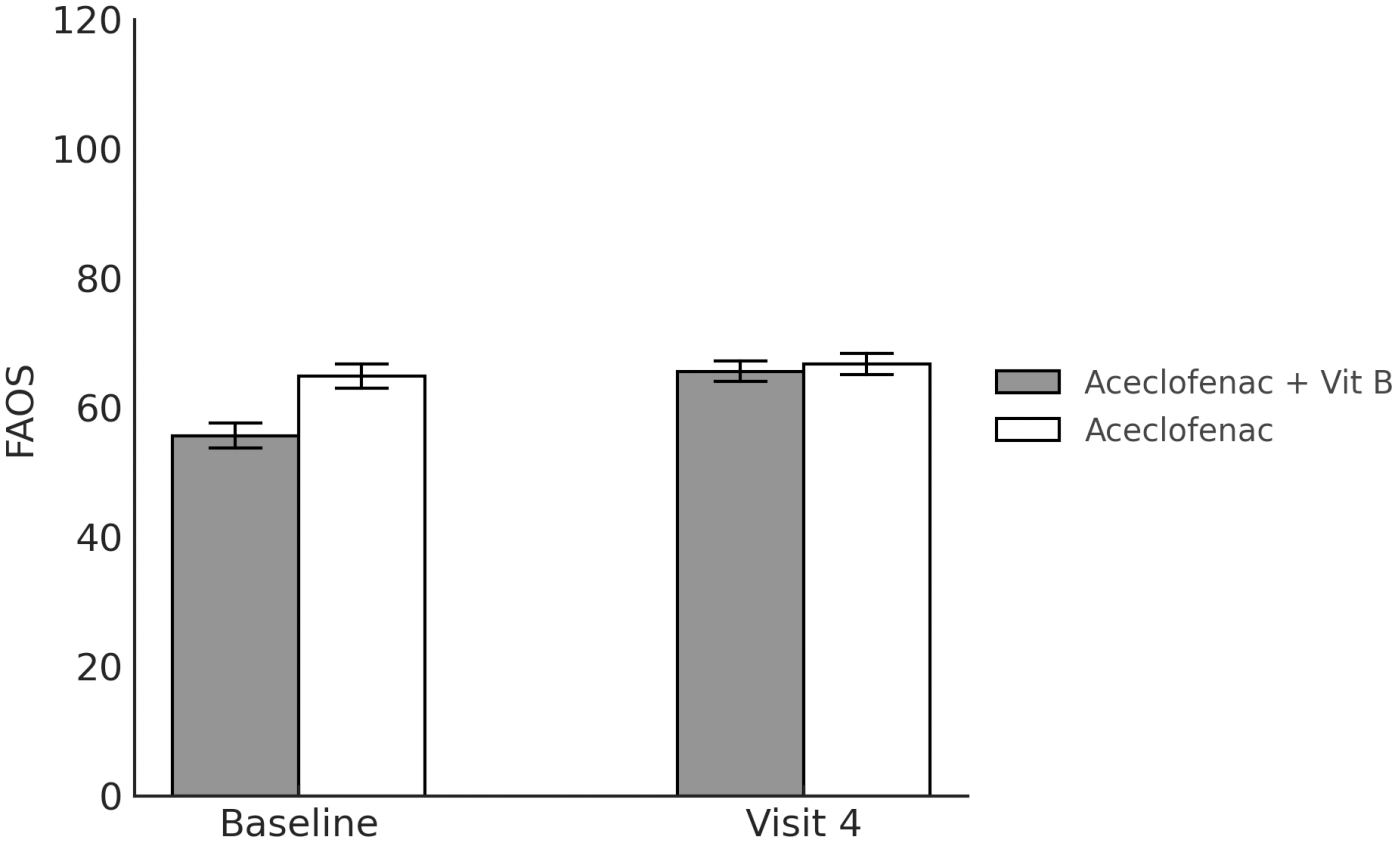
Foot and Ankle Outcome Score at each measurement timepoint.

VAS scores were significantly decreased in both the experimental group (from a pre-treatment average of 6.95 ± 0.15 to 4.08 ± 0.17 after 4 weeks of treatment, p < 0.001) and the control group (from a pre-treatment average of 5.99 ± 0.14

to 5.08 ± 0.21 after 4 weeks of treatment, p < 0.001). Meanwhile, although the EQ-5D scores were improved in both the experimental group (from 0.69 ± 0.01 before treatment to 0.77 ± 0.01 after 4 weeks of treatment, p < 0.001) and in the control group (from 0.75 ± 0.01 before treatment to 0.76 ± 0.01 after 4 weeks of treatment, p = 0.509), the improvement was only significant in the experimental group. With respect to the FAOS, it was significantly improved in both the experimental group (from 55.68 ± 1.95 before treatment to 65.62 ± 1.56 after 4 weeks of treatment, p < 0.001) and the control group (from 64.88 ± 1.88 before treatment to 66.73 ± 1.62 after 4 weeks of treatment, p = 0.030). The outcome measures are shown in Table 4, and Figs 2–4).

Linear mixed modeling revealed a significant time X group interaction for all three outcomes(p < 0.001), indicating that symptom improvement over time was significantly greater in the experimental group.

**Fig 5:**
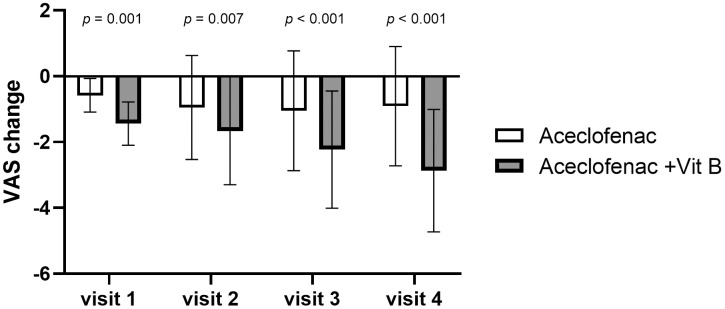
Change in visual analog scale score from baseline to each measurement timepoint.

When the change in VAS scores from baseline to 4 weeks after treatment was compared between the groups, the results showed a larger decrease in the experimental group than in the control group, and the difference was significant (2.87 ± 1.86 vs 0.91 ± 1.81, p < 0.001) (Table 5, Fig 5).

When the changes in the secondary outcome measures from baseline to after 4 weeks of treatment were analyzed, the results showed larger improvements in the EQ-5D score (0.079 ± 0.143 vs. 0.008 ± 0.111, p = 0.001) and FAOS (9.94 ± 14.38 vs. 2.97 ± 11.79, p = 0.001) (Tables 6 and 7). Additional detailed data are provided in the Supporting Information (S1 Table and S2 Table).

### Comparison of VAS scores by condition

The changes in VAS scores between the experimental and control groups were compared for each condition. Among patients with Achilles tendinitis, the VAS scores of the experimental group changed from 7.14 ± 1.68 at baseline to 4.43 ± 2.23 after 4 weeks, whereas those of the control group changed from 6.64 ± 1.69 to 5.82 ± 2.44. The change in VAS scores was not significantly different among the patients with Achilles tendinitis (−2.71 ± 2.69 vs. −0.82 ± 2.75, p = 0.17). For patients with foot and ankle arthritis, the VAS scores of the experimental group changed from 6.81 ± 1.44 at baseline to 4.19 ± 1.56 after 4 weeks, whereas those of the control group changed from 5.55 ± 1.32 to 5.25 ± 1.71, with a significant difference between the two groups (−2.62 ± 1.83 vs. −0.30 ± 1.03, p < 0.001). In patients with Civinini-Morton syndrome, the VAS scores of the experimental group changed from 6.87 ± 1.74 at baseline to 4.61 ± 2.01 after 4 weeks, whereas those of the control group changed from 5.83 ± 1.19 to 4.61 ± 2.02, with a significant difference between the two groups (−3.00 ± 2.00 vs. −1.22 ± 1.95, p = 0.004). For patients with plantar fasciitis, VAS scores changed from 7.07 ± 1.27 at baseline to 4.07 ± 1.76 after 4 weeks, whereas those of the control group changed from 6.22 ± 1.20 to 5.04 ± 1.61, with a significant difference between the two groups (−3.00 ± 1.61 vs. −1.17 ± 1.64, p < 0.001) (Tables 8 and 10). This disease-specific comparison was exploratory and based on unadjusted vas change values, analyzed using independent t-tests.

### Comparison of VAS scores by disease duration

Among patients with a disease duration of less than 3 months, the VAS scores of the experimental group changed from 6.29 ± 1.38 at baseline to 3.71 ± 1.38 after 4 weeks, whereas those of the control group changed from 6.45 ± 1.51 to 4.27 ± 2.15, with no significant difference between the two groups (−2.57 ± 1.61 vs. −2.18 ± 2.89, p = 0.751). For patients with chronic condition (i.e., disease duration exceeding 3 months), the reduction in VAS score was significantly greater in the experimental group than in the control group (Tables 9 and 10). These analyses were exploratory and based on unadjusted change scores. Although the chronic group had a lower baseline VAS, no formal statistical correction was applied.

### Adverse effects

During the 4-week study period, a total of 11 patients discontinued treatment due to adverse effects: 5 in the experimental group (Aceclofenac and vitamin B complex) and 6 in the control group (Aceclofenac alone). The most commonly reported adverse events were dyspepsia, followed by diarrhea and pruritus. All adverse effects were mild and self-limiting, and no serious adverse events requiring hospitalization or emergency medical intervention occurred (Table 11).

**Table 11:** Summary of treatment-related adverse events and withdrawals in each group during the study period.

Adverse effects	Experimental group (aceclofenac + vitB complex, n = 79)	Control group (aceclofenac alone, n = 77)	Total(n = 11)
** Dyspepsia**	3	4	7
** Diarrhea**	2	1	3
** Pruritus**	0	1	1

## Discussion

### Statements and clinical findings

Chronic foot disorders, such as plantar fasciitis, Civinini-Morton syndrome, foot and ankle arthritis, and Achilles tendinitis, are often difficult to treat effectively owing to the feet’s constant weight-bearing role and extensive use in daily life. Consequently, long-term anti-inflammatory drug use is often required, raising concerns about potential side effects. This study shows that patients with foot disorders who take aceclofenac combined with a vitamin B complex experience superior pain relief (as assessed using VAS scores), functional improvement (as assessed using FAOS), and overall health status improvement (as assessed using EQ-5D scores) in comparison to those who take aceclofenac alone. The enhanced pain relief observed in the experimental group receiving vitamin B complex improves the quality of life and potentially reduces the need for prolonged anti-inflammatory drug use.

In the comparison by condition type, the combination of vitamin B complex and aceclofenac results in significantly better pain control than does aceclofenac alone for plantar fasciitis, foot and ankle arthritis, and Civinini-Morton syndrome. For Achilles tendinitis, although the reduction in VAS scores was greater in the experimental group than in the control group (−2.71 ± 2.69 vs. −0.82 ± 2.75, p = 0.17), the difference is not significant. However, this is likely influenced by the small number of patients in this group.

The analysis based on disease duration shows no significant difference in VAS score changes among patients with non-chronic conditions (i.e., lasting less than 3 months). However, in patients with chronic conditions (i.e., disease duration exceeded 3 months), the combination of vitamin B complex and aceclofenac shows a significant synergistic effect for pain control. This indicates that the efficacy for pain control of the combination of an anti-inflammatory drug and vitamin B complex is not significantly different in comparison to that of anti-inflammatory drug monotherapy in patients with non-chronic foot disorders. However, the combination therapy is efficacious for pain control in those with chronic pain lasting ≥3 months.

With respect to safety, mild and self-limiting adverse effects were reported in 11 patients(5 in the experimental group and 6 in the control group), including dyspepsia, diarrhea, and pruritus. No serious treatment-related adverse events occurred. This shows that although the main side effects of anti-inflammatory drugs (e.g.,gastrointestinal and cardiovascular issues) remain a concern, the combination of an anti-inflammatory drug and vitamin B complex for managing foot pain does not significantly increase the risk of side effects. Overall, the findings provide valuable insights into treatment strategies and establish important clinical evidence supporting the efficacy of combination therapy with an anti-inflammatory drug and vitamin B complex for managing foot pain.

Our findings are consistent with previous studies that reported synergistic effects of combining B vitamins with NSAIDs. In the DOLOR study, Mibielli et al. reported that patients with acute low back pain experienced significantly greater pain relief when treated with diclofenac plus a B vitamin complex compared to NSAID monotherapy. Additional randomized trials have further supported this combination approach. Ponce-Monter et al. found that diclofenac combined with B vitamins significantly reduced postoperative pain following lower-limb fracture surgery. Similarly, Magana-Villa et al. showed that a B-vitamin mixture enhanced the analgesic effect of diclofenac in patients with osteoarthritis. Together, these findings highlight the clinical relevance of adjunctive B vitamin therapy in augmenting NSAID efficacy across various musculoskeletal conditions. [11,12,14]

### Pain mechanisms of foot and ankle diseases

The vitamin B complex, particularly vitamin B1 (thiamine), vitamin B6 (pyridoxine), and vitamin B12 (cyanocobalamin), is often referred to as neurotrophic vitamins owing to their essential role in treating neuropathies and their effect on nerve activity [13]. These vitamins demonstrate anti-inflammatory and neuroprotective mechanisms, with each playing distinct roles in various pain and metabolic pathways. They also exert antiallodynic, anti-hyperalgesic, and anti-nociceptive effects [10]. Moreover, in pain management, the concurrent intake of vitamin B complex, particularly neurotrophic vitamins, with analgesics(e.g.,opioids, anticonvulsants, and anti-inflammatory drugs) exhibits a synergistic effect [10].

This study focused on common foot disorders and compared the degree of pain control among patients. Plantar fasciitis, the most common cause of plantar heel pain, results from damage and inflammation of the fascia owing to overuse, leading to both mechanical and neurological pain mechanisms [20]. Achilles tendinitis is an inflammatory condition of the Achilles tendon that, when chronic, progresses to Achilles tendinopathy, leading to degenerative changes in the tendon and involving multiple pain mechanisms [5]. Foot arthritis causes mechanical pain owing to articular lesions and inflammation, activating nociceptors in the joint [21]. Civinini-Morton syndrome, a condition characterized by thickening of the plantar digital nerve at the bifurcation, is a type of nerve entrapment syndrome, with mechanical impingement causing neuropathic pain, commonly associated with metatarsalgia [22,23].

Although each foot disorder exhibits different pain mechanisms, the anti-inflammatory and anti-nociceptive properties of the vitamin B complex can enhance the effects of analgesic anti-inflammatory drugs, producing a synergistic response through a complex mechanism [10]. Given its action on both acute and chronic pain, the vitamin B complex can be particularly effective when administered with anti-inflammatory drugs in managing chronic foot disorders [10,13].

### Limitation

This study only analyzed treatment effects over 4 weeks, limiting the interpretation of long-term outcomes. As this study focused on patients with chronic foot disorders, further research is needed to evaluate sustained pain relief and functional improvement beyond the short-term period.

Another limitation is the absence of blinding for both participants and outcome assessors. Because the primary outcomes(VAS, EQ-5D, and FAOS) are self-reported, the lack of blinding may have introduced performance or detection bias. While the use of standardized, smartphone-based PROM collection may have mitigated some bias, the potential influence of subjective perception cannot be completely ruled out.

## Conclusions

The combination of an anti-inflammatory drug and vitamin B complex demonstrated superior pain control and functional improvement in comparison to an anti-inflammatory drug alone for the treatment of chronic foot disorders. However, further research is needed to confirm the long-term benefits of this combination therapy.

## Supporting information

S1 Table Comparison of baseline characteristics between the experimental and control groups. (DOCX)

S2 Table Comparison of outcome measures between the two groups. (DOCX)

S1 Appendix (XLSX)

S1 File CONSORT-2010-Checklist. (DOCX)

S2 File Protocol. (DOCX)
