# Supplementary material for: Vitamin B supplementation enhances the efficacy of non-steroidal anti-inflammatory drugs in patients with painful foot and ankle conditions: A multicenter, prospective, randomized controlled trial
Source: PLoS One. 2025 Nov 13;20(11):e0336373. doi: 10.1371/journal.pone.0336373 (PMC12614615; doi:10.1371/journal.pone.0336373)
Supplement: S1 Table — (DOCX) [file pone.0336373.s001.docx]

| **Group** Control: 1 Experimental: 2 | **Age** | **Height (cm)** | **Weight (Kg)** | **BMI** | **Disease** Achilles tendinitis: 1 Ankle/foot OA: 2 Morton neuroma: 3 Plantar fasciitis: 4 | **Duration of disease** Within 3m: 1 3-6m: 2 6m-1y: 3 1-2y: 4 Over 2y: 5 | **DM** None: 0 With out neuropathy: 1 | **Alcohol** None: 0 Without neuropathy: 1  Social: 2 | **Smoking** None: 0 Current: 1 Previous: 2 | **Circulation** Abnormal: 0 Normal: 1 |
| --- | --- | --- | --- | --- | --- | --- | --- | --- | --- | --- |
| 1 | 57 | 188.00 | 112.00 | 31.689 | 1 | 3 | 0 | 2 | 0 | 1 |
| 1 | 54 | 153.00 | 51.00 | 21.786 | 3 | 1 | 0 | 0 | 0 | 1 |
| 1 | 64 | 160.00 | 65.00 | 25.391 | 4 | 2 | 1 | 0 | 0 | 1 |
| 1 | 65 | 164.00 | 68.00 | 25.283 | 2 | 1 | 0 | 2 | 0 | 1 |
| 1 | 32 | 180.00 | 90.00 | 27.778 | 1 | 5 | 0 | 0 | 1 | 1 |
| 1 | 69 | 145.00 | 50.00 | 23.781 | 4 | 2 | 0 | 0 | 0 | 1 |
| 1 | 57 | 161.00 | 70.00 | 27.005 | 3 | 3 | 0 | 0 | 0 | 1 |
| 1 | 21 | 155.00 | 46.00 | 19.147 | 2 | 4 | 0 | 0 | 0 | 1 |
| 1 | 57 | 160.00 | 58.00 | 22.656 | 2 | 4 | 0 | 0 | 0 | 1 |
| 1 | 60 | 149.00 | 58.00 | 26.125 | 4 | 2 | 0 | 0 | 0 | 1 |
| 1 | 55 | 153.00 | 58.00 | 24.777 | 3 | 1 | 0 | 0 | 0 | 1 |
| 1 | 59 | 175.00 | 73.00 | 23.840 | 1 | 5 | 0 | 0 | 2 | 1 |
| 1 | 41 | 177.00 | 81.00 | 25.855 | 4 | 1 | 0 | 2 | 1 | 1 |
| 1 | 65 | 165.00 | 63.00 | 23.140 | 3 | 4 | 0 | 0 | 0 | 1 |
| 1 | 56 | 159.00 | 62.00 | 24.524 | 1 | 2 | 0 | 0 | 0 | 1 |
| 1 | 44 | 170.00 | 71.00 | 24.567 | 4 | 2 | 0 | 0 | 0 | 1 |
| 1 | 55 | 160.00 | 57.00 | 22.266 | 4 | 5 | 0 | 0 | 0 | 1 |
| 1 | 29 | 155.00 | 70.00 | 29.136 | 2 | 2 | 0 | 0 | 0 | 1 |
| 1 | 64 | 159.00 | 64.00 | 25.315 | 2 | 4 | 0 | 0 | 0 | 1 |
| 1 | 65 | 155.00 | 56.00 | 23.309 | 4 | 5 | 1 | 0 | 0 | 1 |
| 1 | 59 | 168.00 | 72.00 | 25.510 | 1 | 1 | 0 | 2 | 2 | 1 |
| 1 | 55 | 174.00 | 84.00 | 27.745 | 3 | 1 | 0 | 2 | 0 | 1 |
| 1 | 24 | 180.00 | 88.00 | 27.160 | 2 | 3 | 0 | 2 | 1 | 1 |
| 1 | 44 | 172.00 | 73.00 | 24.676 | 4 | 1 | 0 | 2 | 0 | 1 |
| 1 | 57 | 166.00 | 62.00 | 22.500 | 3 | 2 | 0 | 2 | 0 | 1 |
| 1 | 41 | 170.00 | 70.00 | 24.221 | 4 | 5 | 0 | 2 | 1 | 1 |
| 1 | 20 | 180.00 | 102.00 | 31.481 | 1 | 5 | 0 | 2 | 0 | 1 |
| 1 | 58 | 162.00 | 61.00 | 23.243 | 3 | 3 | 0 | 0 | 1 | 1 |
| 1 | 71 | 174.00 | 71.00 | 23.451 | 3 | 4 | 0 | 0 | 0 | 1 |
| 1 | 43 | 162.00 | 60.00 | 22.862 | 4 | 5 | 0 | 2 | 0 | 1 |
| 1 | 62 | 154.00 | 49.50 | 20.872 | 1 | 1 | 0 | 0 | 0 | 1 |
| 1 | 72 | 160.00 | 72.00 | 28.125 | 3 | 3 | 0 | 0 | 0 | 1 |
| 1 | 29 | 159.00 | 47.00 | 18.591 | 4 | 5 | 0 | 0 | 0 | 1 |
| 1 | 58 | 160.00 | 63.00 | 24.609 | 4 | 4 | 0 | 2 | 0 | 1 |
| 1 | 66 | 173.00 | 73.00 | 24.391 | 2 | 3 | 1 | 2 | 1 | 1 |
| 1 | 38 | 162.50 | 84.00 | 31.811 | 4 | 4 | 0 | 0 | 0 | 1 |
| 1 | 67 | 158.00 | 58.00 | 23.233 | 4 | 2 | 1 | 0 | 0 | 1 |
| 1 | 67 | 170.00 | 75.00 | 25.952 | 2 | 3 | 0 | 0 | 0 | 1 |
| 1 | 65 | 168.00 | 69.00 | 24.447 | 1 | 3 | 0 | 2 | 0 | 1 |
| 1 | 66 | 158.00 | 52.00 | 20.830 | 3 | 3 | 0 | 0 | 0 | 1 |
| 1 | 68 | 168.00 | 58.00 | 20.550 | 2 | 3 | 1 | 2 | 1 | 1 |
| 1 | 73 | 168.00 | 61.00 | 21.613 | 2 | 3 | 1 | 2 | 2 | 1 |
| 1 | 61 | 170.00 | 83.00 | 28.720 | 2 | 5 | 0 | 0 | 0 | 1 |
| 1 | 75 | 173.00 | 73.00 | 24.391 | 2 | 4 | 1 | 2 | 0 | 1 |
| 1 | 65 | 170.00 | 65.00 | 22.491 | 2 | 4 | 0 | 0 | 0 | 1 |
| 1 | 55 | 147.00 | 45.00 | 20.825 | 3 | 1 | 0 | 0 | 0 | 1 |
| 1 | 66 | 148.00 | 50.00 | 22.827 | 4 | 3 | 0 | 0 | 0 | 1 |
| 1 | 50 | 158.00 | 62.00 | 24.836 | 1 | 2 | 0 | 2 | 0 | 1 |
| 1 | 60 | 170.00 | 71.00 | 24.567 | 1 | 4 | 0 | 2 | 0 | 1 |
| 1 | 62 | 165.00 | 57.00 | 20.937 | 1 | 5 | 0 | 2 | 0 | 1 |
| 1 | 50 | 156.00 | 53.00 | 21.778 | 4 | 2 | 0 | 2 | 0 | 1 |
| 1 | 63 | 159.00 | 67.00 | 26.502 | 3 | 5 | 0 | 0 | 0 | 1 |
| 1 | 49 | 159.00 | 73.00 | 28.875 | 4 | 2 | 0 | 0 | 0 | 1 |
| 1 | 62 | 159.00 | 54.00 | 21.360 | 3 | 3 | 0 | 0 | 0 | 1 |
| 1 | 68 | 166.00 | 62.00 | 22.500 | 2 | 5 | 0 | 0 | 0 | 1 |
| 1 | 70 | 170.00 | 66.00 | 22.837 | 4 | 4 | 0 | 2 | 0 | 1 |
| 1 | 60 | 163.00 | 63.00 | 23.712 | 3 | 5 | 0 | 2 | 0 | 1 |
| 1 | 26 | 162.00 | 49.00 | 18.671 | 4 | 4 | 0 | 0 | 0 | 1 |
| 1 | 54 | 160.00 | 51.00 | 19.922 | 3 | 5 | 0 | 0 | 0 | 1 |
| 1 | 48 | 163.00 | 69.00 | 25.970 | 3 | 2 | 0 | 2 | 0 | 1 |
| 1 | 56 | 162.00 | 49.00 | 18.671 | 3 | 4 | 0 | 0 | 0 | 1 |
| 1 | 76 | 153.00 | 55.00 | 23.495 | 2 | 5 | 0 | 0 | 0 | 1 |
| 1 | 73 | 153.00 | 65.00 | 27.767 | 3 | 5 | 0 | 0 | 0 | 1 |
| 1 | 60 | 163.00 | 70.00 | 26.346 | 4 | 2 | 0 | 0 | 0 | 1 |
| 1 | 62 | 158.00 | 43.00 | 17.225 | 2 | 4 | 0 | 0 | 0 | 1 |
| 1 | 65 | 170.00 | 70.00 | 24.221 | 3 | 5 | 0 | 2 | 2 | 1 |
| 1 | 61 | 158.00 | 53.00 | 21.231 | 3 | 5 | 0 | 0 | 0 | 1 |
| 1 | 59 | 170.00 | 65.00 | 22.491 | 4 | 4 | 0 | 1 | 1 | 1 |
| 1 | 63 | 156.00 | 64.00 | 26.298 | 2 | 5 | 0 | 0 | 0 | 1 |
| 1 | 59 | 166.00 | 61.00 | 22.137 | 2 | 5 | 0 | 2 | 0 | 1 |
| 1 | 61 | 158.00 | 55.00 | 22.032 | 4 | 1 | 0 | 0 | 0 | 1 |
| 1 | 63 | 157.00 | 59.00 | 23.936 | 3 | 4 | 0 | 2 | 0 | 1 |
| 1 | 59 | 160.00 | 90.00 | 35.156 | 2 | 4 | 0 | 0 | 1 | 1 |
| 1 | 62 | 149.00 | 47.00 | 21.170 | 3 | 4 | 0 | 0 | 0 | 1 |
| 1 | 60 | 176.00 | 70.00 | 22.598 | 4 | 5 | 0 | 2 | 1 | 1 |
| 1 | 63 | 160.00 | 65.00 | 25.391 | 3 | 1 | 0 | 2 | 0 | 1 |
| 1 | 69 | 159.00 | 58.00 | 22.942 | 2 | 5 | 1 | 2 | 2 | 1 |
| 2 | 27 | 176.00 | 71.00 | 22.921 | 2 | 2 | 0 | 0 | 0 | 1 |
| 2 | 68 | 155.00 | 69.00 | 28.720 | 2 | 4 | 0 | 0 | 0 | 1 |
| 2 | 63 | 158.00 | 60.00 | 24.035 | 2 | 2 | 0 | 0 | 0 | 1 |
| 2 | 64 | 154.50 | 60.00 | 25.136 | 3 | 5 | 0 | 0 | 0 | 1 |
| 2 | 62 | 163.00 | 74.00 | 27.852 | 2 | 5 | 0 | 2 | 1 | 1 |
| 2 | 64 | 164.00 | 75.00 | 27.885 | 3 | 3 | 0 | 0 | 0 | 1 |
| 2 | 56 | 176.00 | 72.00 | 23.244 | 3 | 5 | 0 | 0 | 0 | 1 |
| 2 | 41 | 188.00 | 100.00 | 28.293 | 2 | 5 | 1 | 0 | 1 | 1 |
| 2 | 68 | 152.00 | 54.00 | 23.373 | 4 | 3 | 0 | 0 | 0 | 1 |
| 2 | 64 | 160.00 | 69.00 | 26.953 | 3 | 5 | 0 | 0 | 1 | 1 |
| 2 | 59 | 159.00 | 54.00 | 21.360 | 4 | 4 | 0 | 0 | 0 | 1 |
| 2 | 57 | 154.00 | 68.00 | 28.673 | 4 | 2 | 1 | 0 | 0 | 1 |
| 2 | 73 | 158.00 | 69.00 | 27.640 | 4 | 2 | 0 | 2 | 0 | 1 |
| 2 | 44 | 160.00 | 65.00 | 25.391 | 1 | 2 | 0 | 0 | 0 | 1 |
| 2 | 45 | 176.00 | 80.00 | 25.826 | 2 | 4 | 0 | 0 | 0 | 1 |
| 2 | 50 | 158.00 | 56.00 | 22.432 | 3 | 5 | 0 | 0 | 0 | 1 |
| 2 | 38 | 170.00 | 68.00 | 23.529 | 2 | 4 | 0 | 2 | 0 | 1 |
| 2 | 81 | 166.00 | 66.00 | 23.951 | 2 | 1 | 0 | 0 | 0 | 1 |
| 2 | 61 | 177.00 | 80.00 | 25.535 | 4 | 5 | 1 | 0 | 0 | 1 |
| 2 | 58 | 163.00 | 65.00 | 24.465 | 1 | 1 | 0 | 2 | 2 | 1 |
| 2 | 33 | 163.00 | 70.00 | 26.346 | 4 | 3 | 0 | 2 | 0 | 1 |
| 2 | 73 | 174.00 | 77.00 | 25.433 | 2 | 5 | 0 | 0 | 2 | 1 |
| 2 | 25 | 179.00 | 81.00 | 25.280 | 2 | 1 | 0 | 2 | 0 | 1 |
| 2 | 53 | 164.00 | 64.00 | 23.795 | 3 | 3 | 0 | 2 | 0 | 1 |
| 2 | 63 | 163.00 | 60.00 | 22.583 | 4 | 3 | 0 | 2 | 1 | 1 |
| 2 | 24 | 178.00 | 96.00 | 30.299 | 1 | 2 | 0 | 2 | 1 | 1 |
| 2 | 59 | 170.00 | 60.00 | 20.761 | 2 | 5 | 1 | 2 | 2 | 1 |
| 2 | 58 | 154.00 | 53.00 | 22.348 | 4 | 2 | 0 | 2 | 0 | 1 |
| 2 | 29 | 180.00 | 120.00 | 37.037 | 3 | 4 | 0 | 0 | 1 | 1 |
| 2 | 51 | 163.00 | 78.00 | 29.358 | 1 | 5 | 0 | 2 | 0 | 1 |
| 2 | 21 | 152.70 | 72.70 | 31.179 | 4 | 4 | 0 | 2 | 0 | 1 |
| 2 | 37 | 162.00 | 55.00 | 20.957 | 4 | 5 | 0 | 0 | 0 | 1 |
| 2 | 59 | 165.00 | 70.00 | 25.712 | 3 | 5 | 0 | 2 | 2 | 1 |
| 2 | 37 | 175.00 | 90.00 | 29.388 | 4 | 5 | 0 | 2 | 0 | 1 |
| 2 | 66 | 150.00 | 58.00 | 25.778 | 4 | 4 | 0 | 2 | 0 | 1 |
| 2 | 48 | 164.00 | 65.00 | 24.167 | 4 | 5 | 0 | 0 | 0 | 1 |
| 2 | 64 | 156.00 | 62.00 | 25.477 | 2 | 5 | 0 | 0 | 0 | 1 |
| 2 | 63 | 164.00 | 66.00 | 24.539 | 2 | 4 | 0 | 2 | 2 | 1 |
| 2 | 63 | 159.00 | 73.00 | 28.875 | 2 | 5 | 0 | 0 | 0 | 1 |
| 2 | 70 | 161.00 | 64.00 | 24.690 | 3 | 4 | 0 | 0 | 0 | 1 |
| 2 | 74 | 157.00 | 58.00 | 23.530 | 4 | 3 | 0 | 0 | 0 | 1 |
| 2 | 25 | 165.00 | 75.00 | 27.548 | 1 | 3 | 0 | 0 | 0 | 1 |
| 2 | 20 | 180.00 | 88.00 | 27.160 | 4 | 4 | 0 | 2 | 0 | 1 |
| 2 | 67 | 167.00 | 55.00 | 19.721 | 3 | 2 | 0 | 2 | 0 | 1 |
| 2 | 62 | 158.00 | 63.00 | 25.236 | 2 | 5 | 0 | 0 | 0 | 1 |
| 2 | 53 | 166.00 | 64.00 | 23.225 | 4 | 3 | 0 | 0 | 0 | 1 |
| 2 | 66 | 150.00 | 53.00 | 23.556 | 4 | 4 | 0 | 0 | 0 | 1 |
| 2 | 58 | 165.00 | 63.00 | 23.140 | 3 | 2 | 0 | 0 | 0 | 1 |
| 2 | 52 | 150.00 | 47.00 | 20.889 | 3 | 4 | 0 | 2 | 0 | 1 |
| 2 | 60 | 154.00 | 56.00 | 23.613 | 4 | 2 | 0 | 0 | 0 | 1 |
| 2 | 54 | 161.00 | 68.00 | 26.234 | 3 | 5 | 0 | 2 | 0 | 1 |
| 2 | 70 | 152.00 | 49.00 | 21.208 | 2 | 1 | 0 | 0 | 0 | 1 |
| 2 | 58 | 158.00 | 53.00 | 21.231 | 3 | 5 | 0 | 2 | 0 | 1 |
| 2 | 48 | 160.00 | 61.00 | 23.828 | 4 | 3 | 0 | 0 | 0 | 1 |
| 2 | 52 | 169.00 | 67.00 | 23.459 | 3 | 2 | 0 | 2 | 0 | 1 |
| 2 | 48 | 167.00 | 76.00 | 27.251 | 1 | 3 | 0 | 2 | 0 | 1 |
| 2 | 67 | 150.00 | 70.00 | 31.111 | 2 | 1 | 0 | 0 | 0 | 1 |
| 2 | 52 | 168.00 | 61.00 | 21.613 | 4 | 2 | 0 | 0 | 0 | 1 |
| 2 | 69 | 164.00 | 65.00 | 24.167 | 3 | 4 | 0 | 0 | 0 | 1 |
| 2 | 67 | 165.00 | 58.00 | 21.304 | 4 | 2 | 0 | 0 | 0 | 1 |
| 2 | 53 | 158.00 | 63.00 | 25.236 | 3 | 3 | 0 | 0 | 0 | 1 |
| 2 | 35 | 176.00 | 82.00 | 26.472 | 3 | 5 | 0 | 2 | 1 | 1 |
| 2 | 68 | 165.00 | 76.00 | 27.916 | 4 | 1 | 1 | 2 | 2 | 1 |
| 2 | 64 | 157.00 | 62.00 | 25.153 | 3 | 4 | 0 | 0 | 0 | 1 |
| 2 | 66 | 158.00 | 52.00 | 20.830 | 4 | 5 | 0 | 0 | 0 | 1 |
| 2 | 61 | 158.00 | 68.00 | 27.239 | 2 | 3 | 0 | 0 | 0 | 1 |
| 2 | 55 | 151.00 | 53.00 | 23.245 | 4 | 4 | 0 | 0 | 0 | 1 |
| 2 | 63 | 163.00 | 65.00 | 24.465 | 4 | 4 | 0 | 0 | 1 | 1 |
| 2 | 49 | 158.00 | 60.00 | 24.035 | 4 | 5 | 0 | 2 | 1 | 1 |
| 2 | 57 | 160.00 | 62.00 | 24.219 | 3 | 2 | 0 | 0 | 0 | 1 |
| 2 | 68 | 149.00 | 57.00 | 25.675 | 2 | 5 | 0 | 0 | 0 | 1 |
| 2 | 71 | 165.00 | 70.00 | 25.712 | 3 | 5 | 0 | 2 | 2 | 1 |
| 2 | 51 | 160.00 | 55.00 | 21.484 | 1 | 2 | 0 | 2 | 0 | 1 |
| 2 | 68 | 178.00 | 83.00 | 26.196 | 2 | 5 | 0 | 1 | 2 | 1 |
| 2 | 51 | 173.00 | 72.00 | 24.057 | 4 | 1 | 0 | 2 | 1 | 1 |
| 2 | 67 | 161.00 | 55.00 | 21.218 | 4 | 5 | 1 | 0 | 0 | 1 |
| 2 | 73 | 172.00 | 72.00 | 24.337 | 3 | 5 | 0 | 2 | 2 | 1 |
| 2 | 46 | 170.00 | 80.00 | 27.682 | 2 | 3 | 0 | 0 | 0 | 1 |
| 2 | 60 | 163.00 | 70.00 | 26.346 | 3 | 3 | 1 | 0 | 0 | 1 |
