# Supplementary material for: Vitamin B supplementation enhances the efficacy of non-steroidal anti-inflammatory drugs in patients with painful foot and ankle conditions: A multicenter, prospective, randomized controlled trial
Source: PLoS One. 2025 Nov 13;20(11):e0336373. doi: 10.1371/journal.pone.0336373 (PMC12614615; doi:10.1371/journal.pone.0336373)
Supplement: S2 Table — (DOCX) [file pone.0336373.s002.docx]

| Group | Initial | | | | Visit 1 | | | Visit 2 | | | Visit 3 | | | Last F/U (Visit 4) | | | |
| --- | --- | --- | --- | --- | --- | --- | --- | --- | --- | --- | --- | --- | --- | --- | --- | --- | --- |
|  | EQ-5D-3L | | FAOS | VAS(FOOT) | EQ-5D-3L | | VAS(FOOT) | EQ-5D-3L | | VAS(FOOT) | EQ-5D-3L | | VAS(FOOT) | EQ-5D-3L | | FAOS | VAS(FOOT) |
|  | EQ VAS | EQ-5D Index | Total | Total | EQ VAS | EQ-5D Index | Total | EQ VAS | EQ-5D Index | Total | EQ VAS | EQ-5D Index | Total | EQ VAS | EQ-5D Index | Total | Total |
| 1 | 80 | 0.91 | 79.63 | 4 | 69 | 0.77 | 3 | 80 | 0.95 | 2 | 90 | 0.82 | 3 | 80 | 0.82 | 75.63 | 3 |
| 1 | 85 | 0.91 | 82.35 | 6 | 88 | 0.91 | 6 | 80 | 0.91 | 6 | 85 | 0.91 | 5 | 85 | 0.91 | 78.44 | 5 |
| 1 | 60 | 0.72 | 50 | 6 | 54 | 0.72 | 6 | 50 | 0.72 | 5 | 28 | 0.68 | 4 | 29 | 0.56 | 44.04 | 6 |
| 1 | 50 | 0.72 | 51.3 | 5 | 61 | 0.51 | 6 | 41 | 0.56 | 4 | 30 | 0.72 | 3 | 20 | 0.72 | 62.6 | 4 |
| 1 | 30 | 0.56 | 40.37 | 8 | 63 | 0.72 | 6 | 57 | 0.72 | 6 | 50 | 0.56 | 9 | 30 | 0.4 | 34.78 | 9 |
| 1 | 68 | 0.72 | 41.61 | 5 | 71 | 0.72 | 5 | 66 | 0.72 | 5 | 70 | 0.72 | 7 | 60 | 0.72 | 43.04 | 7 |
| 1 | 51 | 0.72 | 61.24 | 5 | 30 | 0.77 | 3 | 20 | 0.82 | 2 | 10 | 0.77 | 2 | 8 | 0.91 | 94.58 | 1 |
| 1 | 50 | 0.56 | 45.15 | 7 | 80 | 0.72 | 7 | 75 | 0.72 | 7 | 77 | 0.72 | 8 | 75 | 0.72 | 60.67 | 8 |
| 1 | 62 | 0.77 | 86.52 | 4 | 58 | 0.95 | 4 | 61 | 0.76 | 4 | 72 | 0.91 | 4 | 59 | 0.77 | 89.3 | 4 |
| 1 | 56 | 0.72 | 46.3 | 6 | 54 | 0.68 | 5 | 51 | 0.68 | 6 | 53 | 0.68 | 6 | 62 | 0.68 | 42.29 | 6 |
| 1 | 63 | 0.72 | 73.86 | 7 | 70 | 0.72 | 6 | 70 | 0.72 | 6 | 70 | 0.72 | 5 | 50 | 0.82 | 80.62 | 6 |
| 1 | 40 | 0.77 | 53.82 | 5 | 32 | 0.68 | 5 | 30 | 0.72 | 3 | 41 | 0.68 | 3 | 38 | 0.68 | 52.43 | 4 |
| 1 | 60 | 0.68 | 63.24 | 6 | 71 | 0.68 | 6 | 62 | 0.72 | 6 | 50 | 0.68 | 5 | 50 | 0.68 | 62.21 | 5 |
| 1 | 51 | 0.82 | 87.54 | 5 | 40 | 0.72 | 4 | 68 | 0.77 | 3 | 30 | 0.77 | 3 | 36 | 0.72 | 53.29 | 4 |
| 1 | 50 | 0.56 | 54.02 | 9 | 69 | 0.56 | 9 | 69 | 0.56 | 9 | 60 | 0.56 | 7 | 70 | 0.82 | 37.54 | 7 |
| 1 | 61 | 0.77 | 63.38 | 8 | 74 | 0.72 | 7 | 70 | 0.77 | 7 | 51 | 0.77 | 5 | 62 | 0.72 | 66 | 7 |
| 1 | 49 | 0.77 | 55.73 | 7 | 59 | 0.68 | 6 | 62 | 0.72 | 6 | 54 | 0.68 | 6 | 57 | 0.77 | 72.26 | 6 |
| 1 | 61 | 0.72 | 57.94 | 6 | 51 | 0.77 | 5 | 51 | 0.77 | 6 | 50 | 0.82 | 5 | 41 | 0.77 | 76.75 | 4 |
| 1 | 75 | 0.77 | 75.49 | 5 | 60 | 0.72 | 6 | 70 | 0.82 | 5 | 80 | 0.91 | 4 | 78 | 0.77 | 87.86 | 4 |
| 1 | 70 | 0.72 | 61.17 | 8 | 50 | 0.72 | 6 | 63 | 0.72 | 5 | 58 | 0.72 | 6 | 59 | 0.72 | 62.9 | 6 |
| 1 | 71 | 0.72 | 32.8 | 8 | 61 | 0.68 | 7 | 72 | 0.68 | 7 | 62 | 0.68 | 6 | 61 | 0.68 | 52.48 | 7 |
| 1 | 100 | 0.91 | 93.75 | 8 | 91 | 0.82 | 4 | 99 | 0.82 | 1 | 62 | 0.82 | 1 | 90 | 0.95 | 100 | 0 |
| 1 | 53 | 0.68 | 40.13 | 6 | 59 | 0.68 | 6 | 60 | 0.68 | 6 | 60 | 0.68 | 6 | 57 | 0.68 | 50 | 6 |
| 1 | 34 | 0.82 | 74.85 | 4 | 80 | 0.91 | 3 | 80 | 0.91 | 4 | 81 | 0.91 | 4 | 87 | 0.95 | 85.34 | 3 |
| 1 | 68 | 0.51 | 35.93 | 7 | 68 | 0.72 | 6 | 71 | 0.68 | 7 | 70 | 0.47 | 7 | 67 | 0.68 | 41.77 | 7 |
| 1 | 82 | 0.77 | 72.9 | 8 | 52 | 0.77 | 5 | 52 | 0.72 | 4 | 80 | 0.77 | 4 | 80 | 0.82 | 82.12 | 3 |
| 1 | 80 | 0.82 | 76.92 | 5 | 71 | 0.82 | 5 | 70 | 0.82 | 5 | 35 | 0.77 | 7 | 61 | 0.9 | 86.66 | 5 |
| 1 | 50 | 0.91 | 86.48 | 5 | 72 | 0.72 | 4 | 50 | 0.95 | 4 | 50 | 0.82 | 2 | 70 | 0.91 | 81.12 | 5 |
| 1 | 90 | 0.91 | 92.32 | 5 | 60 | 0.77 | 6 | 81 | 0.87 | 6 | 72 | 0.91 | 6 | 82 | 0.91 | 97.16 | 6 |
| 1 | 75 | 0.91 | 59.94 | 8 | 77 | 0.86 | 8 | 50 | 0.86 | 8 | 70 | 0.86 | 8 | 60 | 0.91 | 50.16 | 8 |
| 1 | 77 | 0.6 | 74.73 | 9 | 21 | 0.77 | 2 | 61 | 0.82 | 2 | 88 | 0.95 | 1 | 88 | 0.91 | 75.46 | 1 |
| 1 | 71 | 0.56 | 50.78 | 7 | 53 | 0.54 | 5 | 55 | 0.68 | 5 | 50 | 0.72 | 5 | 50 | 0.72 | 60.55 | 5 |
| 1 | 73 | 0.95 | 84.36 | 4 | 28 | 0.86 | 6 | 81 | 0.95 | 3 | 81 | 0.95 | 3 | 86 | 0.95 | 95.06 | 2 |
| 1 | 61 | 0.91 | 76.81 | 6 | 61 | 0.91 | 6 | 70 | 0.91 | 7 | 71 | 0.91 | 7 | 72 | 0.91 | 62.41 | 7 |
| 1 | 31 | 0.68 | 53.82 | 5 | 32 | 0.68 | 5 | 30 | 0.72 | 3 | 41 | 0.68 | 3 | 38 | 0.68 | 52.43 | 3 |
| 1 | 50 | 0.72 | 55.35 | 7 | 50 | 0.77 | 6 | 50 | 0.77 | 6 | 70 | 0.77 | 5 | 60 | 0.72 | 67.78 | 4 |
| 1 | 90 | 0.91 | 54.01 | 6 | 80 | 0.95 | 3 | 89 | 0.95 | 4 | 90 | 0.95 | 9 | 80 | 0.95 | 91.49 | 2 |
| 1 | 30 | 0.56 | 23.67 | 8 | 40 | 0.56 | 8 | 50 | 0.62 | 8 | 60 | 0.77 | 8 | 70 | 0.56 | 31.28 | 8 |
| 1 | 62 | 0.72 | 88.8 | 6 | 70 | 0.77 | 6 | 60 | 0.82 | 5 | 61 | 0.77 | 6 | 71 | 0.81 | 86.68 | 7 |
| 1 | 81 | 0.72 | 70.76 | 5 | 81 | 0.68 | 5 | 72 | 0.82 | 3 | 95 | 0.82 | 3 | 82 | 0.72 | 70.32 | 3 |
| 1 | 60 | 0.87 | 94.42 | 5 | 72 | 0.72 | 5 | 82 | 0.77 | 3 | 98 | 0.95 | 0 | 16 | 0.91 | 93.79 | 4 |
| 1 | 69 | 0.91 | 74.74 | 4 | 82 | 0.82 | 4 | 29 | 0.82 | 5 | 63 | 0.82 | 3 | 50 | 0.91 | 78.02 | 3 |
| 1 | 82 | 0.82 | 66.79 | 8 | 81 | 0.72 | 7 | 70 | 0.72 | 8 | 77 | 0.68 | 6 | 56 | 0.68 | 73.93 | 6 |
| 1 | 70 | 0.72 | 60.81 | 8 | 62 | 0.77 | 6 | 55 | 0.77 | 5 | 79 | 0.77 | 8 | 80 | 0.63 | 65.33 | 8 |
| 1 | 80 | 0.85 | 71.48 | 5 | 80 | 0.77 | 3 | 79 | 0.77 | 3 | 81 | 0.77 | 2 | 83 | 0.77 | 74.57 | 5 |
| 1 | 80 | 0.72 | 60.96 | 5 | 51 | 0.77 | 7 | 61 | 0.72 | 6 | 77 | 0.77 | 5 | 69 | 0.72 | 64.78 | 5 |
| 1 | 68 | 0.72 | 52.28 | 5 | 64 | 0.77 | 5 | 64 | 0.77 | 6 | 63 | 0.77 | 4 | 66 | 0.77 | 65.38 | 5 |
| 1 | 53 | 0.91 | 60.87 | 6 | 67 | 0.82 | 5 | 70 | 0.82 | 6 | 72 | 0.91 | 6 | 52 | 0.86 | 76.24 | 9 |
| 1 | 61 | 0.77 | 77.01 | 6 | 50 | 0.77 | 5 | 69 | 0.77 | 5 | 40 | 0.72 | 5 | 70 | 0.72 | 75.38 | 6 |
| 1 | 41 | 0.68 | 44.63 | 7 | 52 | 0.72 | 6 | 50 | 0.77 | 5 | 51 | 0.72 | 6 | 59 | 0.68 | 54.62 | 6 |
| 1 | 81 | 0.91 | 80.71 | 5 | 81 | 0.82 | 4 | 90 | 0.91 | 5 | 90 | 0.91 | 5 | 90 | 0.91 | 77.09 | 4 |
| 1 | 50 | 0.77 | 65.16 | 6 | 39 | 0.77 | 5 | 42 | 0.72 | 5 | 51 | 0.77 | 6 | 30 | 0.77 | 62.28 | 6 |
| 1 | 61 | 0.82 | 56.13 | 6 | 50 | 0.91 | 6 | 41 | 0.91 | 5 | 62 | 0.91 | 4 | 62 | 0.91 | 78.57 | 4 |
| 1 | 49 | 0.91 | 82.47 | 4 | 50 | 0.88 | 4 | 68 | 0.88 | 4 | 68 | 0.88 | 4 | 89 | 0.91 | 88.21 | 1 |
| 1 | 51 | 0.51 | 43.39 | 6 | 73 | 0.56 | 4 | 51 | 0.68 | 6 | 51 | 0.68 | 5 | 63 | 0.51 | 53.95 | 7 |
| 1 | 71 | 0.77 | 70.87 | 7 | 50 | 0.77 | 6 | 61 | 0.77 | 5 | 70 | 0.77 | 4 | 71 | 0.77 | 68.82 | 4 |
| 1 | 49 | 0.77 | 87.53 | 5 | 60 | 0.77 | 4 | 70 | 0.91 | 3 | 69 | 0.91 | 3 | 67 | 0.82 | 82.1 | 3 |
| 1 | 55 | 0.91 | 91.15 | 7 | 75 | 0.91 | 4 | 47 | 0.91 | 5 | 75 | 0.88 | 4 | 65 | 0.95 | 88.82 | 5 |
| 1 | 71 | 0.77 | 56.08 | 7 | 68 | 0.72 | 7 | 76 | 0.82 | 7 | 69 | 0.72 | 6 | 79 | 0.77 | 51 | 7 |
| 1 | 60 | 0.52 | 49.96 | 5 | 71 | 0.56 | 8 | 60 | 0.72 | 6 | 58 | 0.72 | 6 | 40 | 0.72 | 54.36 | 4 |
| 1 | 64 | 0.86 | 64.39 | 6 | 51 | 0.72 | 8 | 51 | 0.56 | 8 | 50 | 0.51 | 7 | 50 | 0.51 | 38.58 | 8 |
| 1 | 100 | 0.82 | 70.86 | 4 | 72 | 0.77 | 2 | 31 | 0.82 | 5 | 30 | 0.82 | 3 | 29 | 0.82 | 79.76 | 3 |
| 1 | 50 | 0.71 | 61.88 | 8 | 19 | 0.72 | 6 | 20 | 0.82 | 6 | 9 | 0.86 | 5 | 29 | 0.77 | 67.31 | 6 |
| 1 | 62 | 0.51 | 51.56 | 7 | 50 | 0.77 | 4 | 62 | 0.77 | 3 | 60 | 0.72 | 5 | 60 | 0.72 | 63.1 | 5 |
| 1 | 49 | 0.51 | 44 | 5 | 59 | 0.4 | 6 | 61 | 0.56 | 6 | 60 | 0.68 | 6 | 59 | 0.68 | 37.65 | 6 |
| 1 | 79 | 0.91 | 82.64 | 4 | 35 | 0.72 | 4 | 82 | 0.77 | 2 | 23 | 0.77 | 3 | 20 | 0.72 | 88.03 | 4 |
| 1 | 64 | 0.77 | 63.69 | 7 | 51 | 0.82 | 5 | 51 | 0.72 | 5 | 50 | 0.72 | 5 | 47 | 0.68 | 65.67 | 6 |
| 1 | 69 | 0.72 | 76.19 | 5 | 69 | 0.68 | 7 | 51 | 0.72 | 6 | 50 | 0.72 | 6 | 61 | 0.72 | 80.29 | 6 |
| 1 | 50 | 0.68 | 48.65 | 5 | 51 | 0.68 | 6 | 50 | 0.68 | 6 | 48 | 0.68 | 5 | 50 | 0.68 | 48.57 | 6 |
| 1 | 88 | 0.77 | 61.11 | 5 | 49 | 0.68 | 5 | 50 | 0.72 | 5 | 51 | 0.68 | 5 | 69 | 0.77 | 59.83 | 5 |
| 1 | 50 | 0.91 | 92.79 | 6 | 50 | 0.72 | 6 | 61 | 0.72 | 5 | 80 | 0.77 | 6 | 53 | 0.72 | 85.07 | 5 |
| 1 | 80 | 0.91 | 69.54 | 5 | 43 | 0.91 | 3 | 39 | 0.87 | 4 | 31 | 0.91 | 3 | 33 | 0.77 | 88.01 | 4 |
| 1 | 80 | 0.82 | 55.26 | 6 | 20 | 0.72 | 8 | 82 | 0.72 | 5 | 80 | 0.72 | 8 | 70 | 0.72 | 56.62 | 7 |
| 1 | 51 | 0.81 | 67.16 | 5 | 41 | 0.68 | 5 | 51 | 0.68 | 4 | 40 | 0.68 | 4 | 40 | 0.68 | 42.27 | 4 |
| 1 | 70 | 0.77 | 87.26 | 6 | 61 | 0.68 | 7 | 62 | 0.68 | 5 | 51 | 0.68 | 6 | 60 | 0.68 | 59.54 | 6 |
| 1 | 50 | 0.35 | 43.88 | 7 | 50 | 0.68 | 8 | 52 | 0.77 | 5 | 50 | 0.72 | 5 | 50 | 0.68 | 52.8 | 6 |
| 1 | 41 | 0.9 | 78.45 | 4 | 41 | 0.91 | 4 | 40 | 0.91 | 4 | 49 | 0.86 | 5 | 41 | 0.9 | 87.83 | 4 |
| 2 | 70 | 0.86 | 79.26 | 6 | 70 | 0.86 | 4 | 81 | 0.86 | 5 | 90 | 0.86 | 5 | 94 | 0.91 | 91.94 | 2 |
| 2 | 29 | 0.72 | 64.43 | 7 | 43 | 0.91 | 4 | 42 | 0.82 | 7 | 63 | 0.77 | 7 | 65 | 0.77 | 74.32 | 4 |
| 2 | 40 | 0.4 | 35.6 | 7 | 82 | 0.68 | 6 | 80 | 0.72 | 2 | 90 | 0.77 | 2 | 80 | 0.72 | 67.14 | 2 |
| 2 | 50 | 0.77 | 53.97 | 7 | 38 | 0.77 | 4 | 51 | 0.82 | 5 | 67 | 0.72 | 6 | 65 | 0.77 | 63.52 | 4 |
| 2 | 51 | 0.72 | 64.29 | 7 | 42 | 0.72 | 5 | 60 | 0.77 | 5 | 80 | 0.72 | 6 | 78 | 0.77 | 79.85 | 4 |
| 2 | 58 | 0.77 | 57.7 | 7 | 50 | 0.91 | 5 | 61 | 0.6 | 7 | 63 | 0.77 | 5 | 81 | 0.77 | 70.86 | 3 |
| 2 | 81 | 0.51 | 33.79 | 8 | 72 | 0.68 | 7 | 62 | 0.68 | 3 | 50 | 0.68 | 3 | 50 | 0.68 | 56.77 | 2 |
| 2 | 51 | 0.44 | 31.27 | 10 | 59 | 0.77 | 10 | 50 | 0.77 | 7 | 30 | 0.77 | 8 | 52 | 0.77 | 48.73 | 7 |
| 2 | 60 | 0.72 | 47.82 | 10 | 82 | 0.77 | 7 | 60 | 0.86 | 7 | 63 | 0.82 | 6 | 72 | 0.82 | 62.08 | 4 |
| 2 | 59 | 0.77 | 53.2 | 9 | 50 | 0.72 | 9 | 39 | 0.82 | 5 | 85 | 0.91 | 2 | 95 | 0.91 | 95.88 | 1 |
| 2 | 43 | 0.77 | 69.76 | 8 | 52 | 0.77 | 4 | 71 | 0.72 | 5 | 80 | 0.82 | 4 | 80 | 0.82 | 73.44 | 4 |
| 2 | 72 | 0.6 | 32.44 | 8 | 61 | 0.51 | 8 | 82 | 0.68 | 8 | 89 | 0.68 | 6 | 100 | 0.68 | 48.14 | 4 |
| 2 | 79 | 0.68 | 40.58 | 7 | 51 | 0.72 | 3 | 52 | 0.72 | 5 | 56 | 0.4 | 3 | 60 | 0.51 | 61.47 | 3 |
| 2 | 40 | 0.51 | 21.35 | 9 | 80 | 0.77 | 9 | 98 | 0.91 | 5 | 98 | 0.91 | 5 | 98 | 0.82 | 59.56 | 3 |
| 2 | 81 | 0.51 | 10.8 | 9 | 38 | 0.36 | 8 | 72 | 0.68 | 8 | 74 | 0.68 | 6 | 79 | 0.77 | 39.11 | 5 |
| 2 | 59 | 0.72 | 73.33 | 6 | 59 | 0.72 | 6 | 75 | 0.82 | 5 | 49 | 0.72 | 4 | 70 | 0.77 | 82.45 | 3 |
| 2 | 80 | 0.91 | 83.51 | 6 | 80 | 0.91 | 2 | 90 | 0.95 | 1 | 90 | 0.95 | 1 | 90 | 0.91 | 81.33 | 1 |
| 2 | 50 | 0.82 | 67.78 | 5 | 60 | 0.68 | 4 | 40 | 0.77 | 5 | 40 | 0.72 | 4 | 30 | 0.72 | 57.12 | 3 |
| 2 | 62 | 0.82 | 81.36 | 7 | 62 | 0.77 | 5 | 63 | 0.77 | 6 | 62 | 0.77 | 7 | 51 | 0.85 | 76.7 | 5 |
| 2 | 82 | 0.51 | 39.38 | 8 | 71 | 0.72 | 5 | 81 | 0.77 | 2 | 82 | 0.72 | 2 | 93 | 0.86 | 65.72 | 2 |
| 2 | 80 | 0.77 | 54.25 | 7 | 81 | 0.58 | 10 | 90 | 0.95 | 4 | 90 | 0.95 | 2 | 90 | 0.95 | 83.55 | 2 |
| 2 | 72 | 0.72 | 62.08 | 7 | 60 | 0.77 | 3 | 62 | 0.72 | 5 | 60 | 0.72 | 5 | 81 | 0.77 | 76.78 | 4 |
| 2 | 73 | 0.77 | 61.8 | 8 | 64 | 0.82 | 6 | 61 | 0.86 | 7 | 65 | 0.77 | 6 | 66 | 0.58 | 70.27 | 6 |
| 2 | 72 | 0.77 | 61.74 | 7 | 62 | 0.68 | 6 | 46 | 0.77 | 5 | 37 | 0.77 | 4 | 33 | 0.77 | 62.24 | 4 |
| 2 | 71 | 0.86 | 85.14 | 6 | 80 | 0.91 | 1 | 80 | 0.91 | 5 | 80 | 0.95 | 1 | 82 | 0.91 | 85.62 | 3 |
| 2 | 72 | 0.77 | 59.3 | 7 | 63 | 0.77 | 6 | 70 | 0.77 | 7 | 80 | 0.77 | 5 | 62 | 0.77 | 67.08 | 6 |
| 2 | 60 | 0.77 | 49.76 | 6 | 60 | 0.86 | 5 | 61 | 0.68 | 6 | 58 | 0.72 | 6 | 60 | 0.72 | 65.49 | 6 |
| 2 | 21 | 0.91 | 68.34 | 8 | 30 | 0.77 | 3 | 45 | 0.77 | 4 | 37 | 0.77 | 4 | 35 | 0.77 | 68.76 | 3 |
| 2 | 80 | 0.82 | 60.01 | 10 | 80 | 0.77 | 10 | 80 | 0.82 | 6 | 80 | 0.82 | 6 | 50 | 0.86 | 72.51 | 7 |
| 2 | 40 | 0.68 | 36.47 | 5 | 59 | 0.51 | 5 | 61 | 0.68 | 6 | 72 | 0.68 | 6 | 72 | 0.51 | 25.84 | 6 |
| 2 | 37 | 0.61 | 48.77 | 8 | 23 | 0.68 | 6 | 19 | 0.72 | 7 | 31 | 0.56 | 8 | 56 | 0.82 | 54.9 | 4 |
| 2 | 75 | 0.77 | 68.92 | 7 | 79 | 0.77 | 6 | 78 | 0.77 | 7 | 70 | 0.77 | 8 | 80 | 0.77 | 74.9 | 7 |
| 2 | 69 | 0.68 | 86.78 | 5 | 50 | 0.72 | 4 | 51 | 0.72 | 5 | 70 | 0.72 | 4 | 49 | 0.72 | 71.91 | 4 |
| 2 | 80 | 0.58 | 46.26 | 8 | 65 | 0.77 | 7 | 60 | 0.77 | 4 | 60 | 0.77 | 4 | 85 | 0.91 | 68.64 | 3 |
| 2 | 60 | 0.77 | 63.01 | 7 | 71 | 0.82 | 7 | 71 | 0.77 | 7 | 68 | 0.86 | 7 | 80 | 0.86 | 75.14 | 7 |
| 2 | 70 | 0.86 | 62.24 | 8 | 50 | 0.82 | 8 | 30 | 0.91 | 6 | 51 | 0.91 | 6 | 55 | 0.91 | 68.49 | 6 |
| 2 | 51 | 0.77 | 54.8 | 4 | 51 | 0.72 | 5 | 42 | 0.72 | 5 | 41 | 0.72 | 5 | 50 | 0.77 | 60.18 | 6 |
| 2 | 90 | 0.77 | 84.56 | 6 | 80 | 0.77 | 0 | 81 | 0.82 | 0 | 80 | 0.68 | 2 | 80 | 0.68 | 78.72 | 2 |
| 2 | 61 | 0.51 | 47.24 | 8 | 52 | 0.51 | 5 | 60 | 0.68 | 6 | 62 | 0.68 | 6 | 56 | 0.68 | 65.6 | 5 |
| 2 | 68 | 0.72 | 56.89 | 8 | 69 | 0.91 | 6 | 81 | 0.72 | 7 | 75 | 0.77 | 5 | 70 | 0.87 | 62.53 | 6 |
| 2 | 50 | 0.56 | 41.78 | 8 | 70 | 0.36 | 7 | 74 | 0.56 | 5 | 65 | 0.72 | 5 | 67 | 0.42 | 45.09 | 6 |
| 2 | 42 | 0.6 | 61.17 | 9 | 50 | 0.56 | 8 | 60 | 0.68 | 8 | 41 | 0.77 | 8 | 80 | 0.56 | 54.01 | 8 |
| 2 | 50 | 0.77 | 36.78 | 7 | 61 | 0.86 | 8 | 59 | 0.91 | 8 | 66 | 0.72 | 7 | 51 | 0.77 | 38.95 | 6 |
| 2 | 93 | 0.68 | 66.73 | 4 | 44 | 0.72 | 4 | 45 | 0.68 | 4 | 47 | 0.68 | 5 | 47 | 0.72 | 85.24 | 5 |
| 2 | 81 | 0.77 | 60.33 | 5 | 80 | 0.77 | 5 | 71 | 0.77 | 5 | 71 | 0.77 | 5 | 71 | 0.72 | 57.81 | 6 |
| 2 | 50 | 0.68 | 51.55 | 6 | 38 | 0.68 | 4 | 49 | 0.68 | 4 | 52 | 0.68 | 4 | 71 | 0.68 | 27.85 | 4 |
| 2 | 59 | 0.39 | 71.72 | 5 | 33 | 0.91 | 3 | 20 | 0.95 | 6 | 21 | 0.87 | 3 | 21 | 0.91 | 96.54 | 2 |
| 2 | 57 | 0.6 | 51.11 | 6 | 60 | 0.72 | 5 | 62 | 0.77 | 5 | 70 | 0.82 | 3 | 69 | 0.82 | 71.89 | 3 |
| 2 | 50 | 0.72 | 38.01 | 7 | 51 | 0.72 | 6 | 61 | 0.72 | 6 | 71 | 0.77 | 6 | 50 | 0.44 | 40.81 | 7 |
| 2 | 80 | 0.77 | 69.4 | 8 | 71 | 0.77 | 7 | 78 | 0.77 | 8 | 60 | 0.77 | 7 | 61 | 0.77 | 61.32 | 7 |
| 2 | 78 | 0.36 | 66.48 | 8 | 80 | 0.56 | 7 | 50 | 0.68 | 7 | 61 | 0.68 | 6 | 50 | 0.68 | 64.17 | 5 |
| 2 | 49 | 0.82 | 68.23 | 5 | 70 | 0.85 | 3 | 80 | 0.77 | 3 | 81 | 0.77 | 3 | 82 | 0.91 | 67.53 | 4 |
| 2 | 51 | 0.72 | 57.17 | 9 | 50 | 0.56 | 5 | 50 | 0.72 | 7 | 60 | 0.77 | 5 | 39 | 0.72 | 44.02 | 4 |
| 2 | 72 | 0.72 | 62.4 | 9 | 69 | 0.72 | 8 | 70 | 0.68 | 8 | 79 | 0.72 | 8 | 81 | 0.68 | 47.78 | 8 |
| 2 | 68 | 0.77 | 71.84 | 4 | 70 | 0.77 | 4 | 38 | 0.77 | 5 | 45 | 0.77 | 5 | 65 | 0.77 | 70.54 | 4 |
| 2 | 50 | 0.72 | 42.29 | 7 | 65 | 0.77 | 5 | 59 | 0.72 | 5 | 69 | 0.72 | 5 | 83 | 0.82 | 53.81 | 3 |
| 2 | 80 | 0.72 | 45.17 | 7 | 70 | 0.77 | 7 | 60 | 0.72 | 6 | 60 | 0.72 | 6 | 55 | 0.77 | 63.31 | 5 |
| 2 | 50 | 0.51 | 16.41 | 8 | 50 | 0.72 | 4 | 58 | 0.72 | 5 | 60 | 0.51 | 3 | 80 | 0.91 | 82.04 | 2 |
| 2 | 50 | 0.77 | 70.6 | 8 | 52 | 0.91 | 5 | 50 | 0.95 | 5 | 50 | 0.95 | 5 | 71 | 0.91 | 85.23 | 4 |
| 2 | 78 | 0.68 | 47.87 | 8 | 39 | 0.72 | 7 | 62 | 0.72 | 6 | 75 | 0.77 | 5 | 78 | 0.82 | 57.69 | 4 |
| 2 | 74 | 0.82 | 82.59 | 6 | 74 | 0.91 | 6 | 40 | 0.82 | 4 | 71 | 0.82 | 3 | 81 | 0.77 | 83.89 | 3 |
| 2 | 70 | 0.86 | 72.36 | 5 | 50 | 0.82 | 5 | 50 | 0.77 | 5 | 40 | 0.77 | 4 | 87 | 0.91 | 78.12 | 3 |
| 2 | 29 | 0.72 | 75.88 | 6 | 81 | 0.72 | 6 | 80 | 0.68 | 4 | 70 | 0.68 | 3 | 84 | 0.77 | 61.63 | 3 |
| 2 | 70 | 0.56 | 31.17 | 9 | 82 | 0.51 | 9 | 71 | 0.68 | 8 | 72 | 0.68 | 7 | 61 | 0.72 | 50.88 | 5 |
| 2 | 73 | 0.52 | 73.61 | 5 | 0 | 0.95 | 0 | 12 | 0.95 | 2 | 15 | 0.91 | 1 | 20 | 0.91 | 80.38 | 2 |
| 2 | 82 | 0.4 | 10.97 | 8 | 20 | -0.08 | 7 | 98 | 0.77 | 6 | 97 | -0.17 | 5 | 74 | 0.51 | 38.83 | 4 |
| 2 | 61 | 0.91 | 72.13 | 5 | 70 | 0.91 | 4 | 50 | 0.82 | 4 | 72 | 0.86 | 4 | 81 | 0.91 | 78.47 | 2 |
| 2 | 70 | 0.77 | 43.82 | 6 | 59 | 0.77 | 5 | 59 | 0.77 | 5 | 62 | 0.91 | 5 | 62 | 0.82 | 63.64 | 3 |
| 2 | 81 | 0.77 | 39.5 | 7 | 61 | 0.68 | 6 | 51 | 0.72 | 5 | 75 | 0.86 | 4 | 51 | 0.85 | 52.88 | 4 |
| 2 | 31 | 0.56 | 35.76 | 9 | 62 | 0.68 | 7 | 61 | 0.72 | 8 | 62 | 0.77 | 6 | 80 | 0.72 | 45.52 | 4 |
| 2 | 70 | 0.68 | 48.95 | 7 | 60 | 0.72 | 6 | 59 | 0.72 | 5 | 77 | 0.82 | 4 | 90 | 0.82 | 88.87 | 4 |
| 2 | 72 | 0.72 | 60.9 | 6 | 61 | 0.77 | 7 | 61 | 0.72 | 7 | 71 | 0.68 | 5 | 81 | 0.86 | 71.68 | 4 |
| 2 | 50 | 0.72 | 71.11 | 5 | 60 | 0.72 | 5 | 40 | 0.77 | 4 | 70 | 0.77 | 4 | 75 | 0.77 | 76.77 | 3 |
| 2 | 50 | 0.68 | 41.57 | 8 | 62 | 0.68 | 6 | 62 | 0.72 | 5 | 73 | 0.77 | 4 | 60 | 0.68 | 55.72 | 4 |
| 2 | 80 | 0.82 | 59.61 | 5 | 82 | 0.58 | 3 | 71 | 0.91 | 4 | 74 | 0.86 | 4 | 70 | 0.86 | 61.91 | 3 |
| 2 | 70 | 0.51 | 24.79 | 6 | 62 | 0.56 | 5 | 58 | 0.15 | 5 | 60 | 0.76 | 5 | 61 | 0.72 | 38.12 | 3 |
| 2 | 50 | 0.72 | 50.38 | 5 | 40 | 0.77 | 4 | 40 | 0.72 | 4 | 40 | 0.72 | 4 | 70 | 0.82 | 68.95 | 3 |
| 2 | 50 | 0.72 | 59.6 | 7 | 73 | 0.77 | 8 | 86 | 0.87 | 4 | 84 | 0.82 | 5 | 85 | 0.91 | 71.09 | 4 |
| 2 | 90 | 0.82 | 87.08 | 5 | 11 | 0.85 | 0 | 91 | 0.95 | 1 | 81 | 0.82 | 0 | 79 | 0.95 | 86.26 | 1 |
