# Supplementary material for: Vitamin B supplementation enhances the efficacy of non-steroidal anti-inflammatory drugs in patients with painful foot and ankle conditions: A multicenter, prospective, randomized controlled trial
Source: PLoS One. 2025 Nov 13;20(11):e0336373. doi: 10.1371/journal.pone.0336373 (PMC12614615; doi:10.1371/journal.pone.0336373)
Supplement: S2 File — (DOCX) [file pone.0336373.s005.docx]

**Title**

Vitamin B supplementation enhances the efficacy of non-steroidal anti-inflammatory drugs in patients with painful foot and ankle conditions: Study Protocol

**IRB Approval**

Inha University Hospital Institutional Review Board(IRB No.2019-11-055, Approved December 2019)

**Trial Registration**

Clinical Research Information Service(CRIS) - KCT0005035(Registered May 19,2020)

**Study Design**

Multicenter, prospective, randomized comparative study, controlled clinical trial. This trial was conducted at four university hospitals in Korea (Inha University hospital, Asan Medical Center, Korea University Ansan Hospital, and Yeungnam University Hospital) between October 2020 and December 2021.

**Study Objectives**

To evaluate whether the combination of a vitamin B complex with a non-steroidal anti-inflammatory drug (NSAID) provides superior pain relief compared to NSAID monotherapy in patients with painful foot and ankle disorders.

**Eligibility Criteria**

The study included patients over 19 years old suffering from one of the four foot disorders(Foot or ankle osteoarthritis, Plantar fasciitis, Achilles tendinitis, Civinini-Morton neuroma) and experiencing a pain score of at least 4cm on the 10cm Visual Analog Scale (VAS) who required NSAIDs for pain control.

**Exclusion criteria**

-Baseline VAS <4 cm

-Use of NSAIDs or other analgesics within the past week

-Presence of open wounds, infection, or planned surgery

-History of depression or cognitive impairment

-Concurrent vitamin supplementation

-Systemic autoimmune disease under treatment

-Inability to understand study procedures or provide consent

**Interventions**

- Experimental group: Aceclofenac 100 mg BID + Vitamin B complex (Impactamin Power®, Daewoong Pharm.) BID for 4 weeks.
- Control group: Aceclofenac 100 mg BID alone for 4 weeks.

**Efficacy Assessments**

- Primary outcome: Change in pain intensity measured by the Visual Analog Scale (VAS) at 4 weeks.
- Secondary outcomes: Changes in EQ-5D and FAOS (Foot and Ankle Outcome Score) from baseline to week 4; incidence of adverse events.

**Sample Size and Statistical Analysis**

The target sample size was 200 participants (100 per group). Sample size was calculated based on the DOLOR study, assuming a 7 mm superiority margin, α = 0.05, and power = 0.9.
Between-group comparisons were performed using ANCOVA adjusted for baseline scores, and repeated measures were analyzed using linear mixed-effects models. Statistical significance was defined as p < 0.05.

**Data Collection and Management**

Data were collected through electronic case report forms (eCRF) using the ProScore system. Participants self-reported VAS, EQ-5D, and FAOS scores weekly via smartphone-based ePRO at baseline, 1, 2, 3, and 4 weeks.

**Safety Assessment**

All adverse events, including mild gastrointestinal discomfort, diarrhea, or pruritus, were recorded and reported to the IRB. No serious drug-related adverse events were expected due to the over-the-counter nature of the vitamin formulation.

**Ethics**

The study was conducted in accordance with the Declaration of Helsinki and Good Clinical Practice (GCP) guidelines. Written informed consent was obtained from all participants. Participant confidentiality was ensured through anonymization of datasets and secure data storage.

**Data Availability**

All data supporting the findings of this study are available in the Supporting Information (S1 Appendix).
